# Passive epidemiological surveillance in wildlife in Costa Rica identifies pathogens of zoonotic and conservation importance

**DOI:** 10.1371/journal.pone.0262063

**Published:** 2022-09-26

**Authors:** Fernando Aguilar-Vargas, Tamara Solorzano-Scott, Mario Baldi, Elías Barquero-Calvo, Ana Jiménez-Rocha, Carlos Jiménez, Marta Piche-Ovares, Gaby Dolz, Bernal León, Eugenia Corrales-Aguilar, Mario Santoro, Alejandro Alfaro-Alarcón

**Affiliations:** 1 Departamento de Patología, Escuela de Medicina Veterinaria, Universidad Nacional, Costa Rica; 2 Servicio Nacional de Salud Animal, Ministerio de Agricultura y Ganadería, Costa Rica; 3 Programa de Investigación en Enfermedades Tropicales, Escuela de Medicina Veterinaria, Universidad Nacional, Costa Rica; 4 Laboratorio de Bacteriología, Escuela de Medicina Veterinaria, Universidad Nacional, Costa Rica; 5 Laboratorio de Parasitología, Escuela de Medicina Veterinaria, Universidad Nacional, Costa Rica; 6 Laboratorio de Virología, Escuela de Medicina Veterinaria, Universidad Nacional, Costa Rica; 7 Laboratorio de Zoonosis y Entomología, Escuela de Medicina Veterinaria, Universidad Nacional, Costa Rica; 8 Faculty of Microbiology, Virology-CIET (Research Center for Tropical Diseases), Universidad de Costa Rica, Costa Rica; 9 Department of Integrative Marine Ecology, Stazione Zoologica Anton Dohrn, Italy; University of Oklahoma Norman Campus: The University of Oklahoma, UNITED STATES

## Abstract

Epidemiological surveillance systems for pathogens in wild species have been proposed as a preventive measure for epidemic events. These systems can minimize the detrimental effects of an outbreak, but most importantly, passive surveillance systems are the best adapted to countries with limited resources. Therefore, this research aimed to evaluate the technical and infrastructural feasibility of establishing this type of scheme in Costa Rica by implementing a pilot program targeting the detection of pathogens of zoonotic and conservation importance in wildlife. Between 2018 and 2020, 85 carcasses of free-ranging vertebrates were admitted for post-mortem and microbiology analysis. However, we encountered obstacles mainly related to the initial identification of cases and limited local logistics capacity. Nevertheless, this epidemiological surveillance scheme allowed us to estimate the general state of health of the country’s wildlife by establishing the causes of death according to pathological findings. For instance, 60% (51/85) of the deaths were not directly associated with an infectious agent. Though in 37.6% (32/85) of these cases an infectious agent associated or not with disease was detected. In 27.1% (23/85) of the cases, death was directly related to infectious agents. Furthermore, 12.9% (11/85), the cause of death was not determined. Likewise, this wildlife health monitoring program allowed the detection of relevant pathogens such as Canine Distemper Virus, *Klebsiella pneumoniae*, *Angiostrongylus* spp., *Baylisascaris* spp., among others. Our research demonstrated that this passive surveillance scheme is cost-effective and feasible in countries with limited resources. This passive surveillance can be adapted to the infrastructure dedicated to monitoring diseases in productive animals according to the scope and objectives of monitoring wildlife specific to each region. The information generated from the experience of the initial establishment of a WHMP is critical to meeting the challenges involved in developing this type of scheme in regions with limited resources and established as hotspots for emerging infectious diseases.

## Introduction

Zoonotic diseases directly threaten public health systems, generating costs in medical treatment, outbreak control, and overloading health systems. In addition, it generates significant losses due to the slaughter of livestock and the affectation of other domestic animals [1,2]. Examples of how these diseases can impact public health, animal health, and wildlife have been the recent outbreaks of yellow fever and West Nile virus, which show the need to have the infrastructure and diagnostic capacity to ensure constant surveillance of potentially zoonotic agents [3,4].

Wildlife populations act as reservoirs and can play various roles in the epidemiology of numerous pathogens [5–7]. These roles assign to wildlife the important function of sentinels of the health of ecosystems and allow early detection of environmental alterations and the distribution, re-emergence, or emergence of certain pathogens in a specific region [8,9].

Tropical regions are among the areas of most extraordinary natural diversity with a concomitant high diversity of pathogens and, thus, a high potential for disease emergence [10,11]. Moreover, this risk has increased drastically because of anthropogenic pressures linked to over-exploitation of natural resources and increased land use change, increasing the possibility of contact between wildlife, domestic animals, and humans [12,13].

One of the preventive strategies against the risk of epidemic events promoted by the World Organization for Animal Health (OIE) and the World Health Organization (WHO) is to increase the efforts to establish early detection mechanisms for pathogens, of both zoonotic and conservation importance, via Wildlife Health Monitoring Programs (WHMP) [14–16].

One of the first steps to knowing the health status of the wildlife in a region is monitoring through passive surveillance, which identifies the causes of mortality in a range of species based on their pathological profiles through post-mortem examinations. This approach offers advantages like cost-effectiveness and the ability to carry out convenience samplings, taking advantage of the established infrastructure and diagnostic capacity. Furthermore, when these schemes are set in the long term, it has been shown that they provide the core information for decision-making and the establishment of policies, norms, and strategies, prioritizing disease prevention, even when the sampling is biased and with incomplete geographic coverage [17–20].

In Latin America have been made some significant efforts to improve epidemiological surveillance systems aimed at animal health. Some national programs are installed and functioning perfectly where wild animals are used as sentinels to monitor specific diseases [21,22]. However, there are still no monitoring programs for the general health status of wildlife, making clear the need to optimize and expand the coverage of these schemes [23,24]. For example, according to the U.S. Department of Agriculture, Costa Rica has the infrastructure and maintains adequate surveillance programs to detect and control zoonotic diseases in livestock [25]. However, it does not contemplate local wildlife within its scheme as it should [26].

Several pathogens, such as zoonotic parasites, vector-borne infectious agents, and direct transmission viruses, have been identified in Costa Rican wildlife [27–38]. This evidence reflects the urgency of establishing a permanent WHMP, where aspects such as general health status and monitoring of zoonotic pathogens in wildlife are considered, facilitating knowledge of the ecoepidemiology of these agents at the local level.

Countries with limited resources, such as Costa Rica, face severe financial and logistical restrictions in monitoring the health and circulation of pathogens in wildlife. Nevertheless, in the short term must extend the coverage of this type of program to tropical regions. Therefore, this research aims to evaluate the technical and infrastructural feasibility of establishing this type of scheme in Costa Rica by implementing a pilot program for passive epidemiological surveillance of wildlife. Although we encountered obstacles such as a lack of data collection legislation and a willingness to cooperate among agencies, our research demonstrated the logistical capacity and that it is possible to adapt the established infrastructure to implement this program. Furthermore, this allowed wild animal carcasses to be analyzed, detecting zoonotic pathogens and pathogens of conservation importance.

## Material and methods

### Statement of ethics

All samples were obtained from dead wildlife (found dead in the field or euthanized after veterinary care in specialized centers). The study was approved by the Ministry of Environment and Energy (MINAE) (wildlife authority) through permits (R-SINAC-PNI: -ACAT-040, ACAHN-18, ACTo-022, ACT-OR-DR-43, ACG-026, ACLAC-039, ACLAP-023, ACOPAC-005, ACC-037), and with the support of the animal health authority, the National Animal Health Service through the office (SENASA-DG-0277-18).

### WHMP schema proposal and case definition

**Fig 1:**
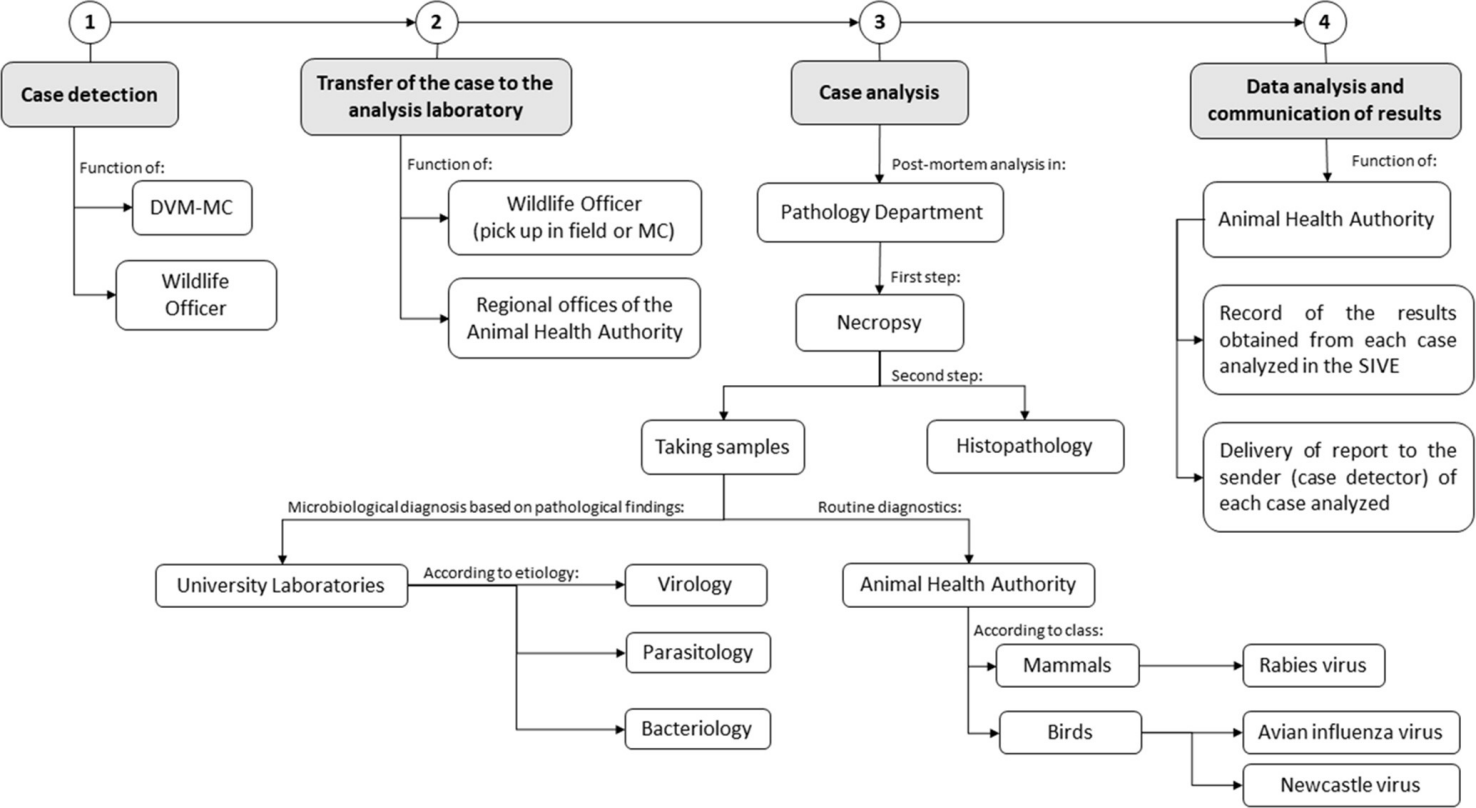
Pilot WHMP work scheme design proposal. DVM-MC: Doctor of veterinary medicine of wildlife management centers; MC: Wildlife management centers; PD: Pathology Department of Escuela de Medicina Veterinaria, Universidad Nacional.

For the implementation of a WHMP, a passive epidemiological surveillance scheme was proposed adapting the current country’s technical diagnostic resources and infrastructure. To create a network for detecting dead and diseased wild animals, officials from the wildlife management centers and officials from wildlife authorities reported cases and voluntarily sent specimens. Officials were encouraged to send complete carcasses from free-ranging vertebrates after death due to any associated disease or trauma, both found dead in the field or deceased in management centers. Carcasses of animals that remained more than 48 hours in the management centers before death, received medication, or were frozen for more than a week were excluded from the study. The proposed WHMP scheme is shown in Fig 1.

Basic information was requested and registered for every sample submission: geographic location, the standard and scientific name of the animal, clinical signs, and any information considered relevant to the case, following the scheme recommended by the OIE for the notification of cases for disease surveillance system in wild animals [16,39]. All carcasses were shipped under refrigerated conditions at 2–8°C.

### Pathological analysis

The carcasses received were classified by autolysis degree according to an established scale of one to five [40]. Thus, ranging from a fresh carcass or recently dead animal (grade 1) to advanced decomposition (grade 4) and partial, mummified carcasses or skeletal remains (grade 5). Only carcasses with grades 1 to 3 were included in the study for post-mortem analysis and tissue sampling [41]. Therefore, 96 specimens were received, of which 85 were admitted to the study. Specimens were divided by sex and age according to the development of sexual organs and phenotypic characteristics of the species. Also, they were divided by taxonomic order.

All morphological findings were recorded. In addition, tissue samples were taken for routine histopathological and microbiological analysis as required. Tissue samples for histopathology were processed based on standard routing protocols [41].

### Detection of different infectious agents

#### Virus detection

Molecular methods were used to detect different viral agents. All molecular methods were done in the presence of positive and negative controls. The samples analyzed were fresh tissues collected sterile during post-mortem analysis. In addition, we performed RNA extraction using the commercial kit DNeasy Blood and Tissue (QIAGEN, Venlo, The Netherlands), following the manufacturer’s recommendations. The methods used and the samples collected are specified in Table 1.

**Table 1:** Molecular techniques for the detection of viral agents and protozoa.

Infectious agent	Target region	Method	Primer	Sequence	Reference protocol	Used material
**Canine Distemper Virus (CDV).**	N gene	Nested RT-PCR	First round: CDV-1F	*5’- ACT GCT CCT GAT ACT GC-3’*	Da Budaszewski et al., 2014. [42]	Tissue^a^
			CDV-2R	*5’- TTC AAC ACC RAC YCC C-3’*		
			Second round: CDV-3F	*5’- ACA GRA TTG CYG AGG ACY TRT-3*’		
			CDV-4R	*5’- CAR RAT AAC CAT GTA YGG TGC-3’*		
**Alphaviruses.**	nsP4	Nested RT-PCR	First round: --	*5’- TTT AAG TTT GGT GCG ATG ATG AAG TC-3’ (500 nM)*	Grywna et al., 2010. [43]	Tissue^a^
				*5’- GCA TCT ATG ATA TTG ACT TCC ATG TT-3’ (500 nM)*		
			Second round: --	*5’-GGT GCG ATG ATG AAG TCT GGG ATG T-3’ (200nM)*		
				*5’- CTA TGA TAT TGA CTT CCA TGT TCA TCC A-3’ (100 nM)*		
				*5’-CTA TGA TAT TGA CTT CCA TGT TCA GCC A-3’ (100 nM)*		
**Flaviviruses.**	NS5 gene	Semi-nested RT-PCR	First round: MAMD	*5’- AAC ATG ATG GGR AAR AGR GAR AA-3’*	Scaramozzino et al., 2001. [44]	Tissue^a^
			cFD2	5’-GTG TCC CAG CCG GCG GTG TCA TCA GC-3’		
			Second round: FS 778	*5’-AAR GGH AGY MCD GCH ATH TGG T-3’*		
			cFD2	*5’-GTG TCC CAG CCG GCG GTG TCA TCA GC-3’*		
**Avian Influenza virus (AI).**	matrix (M) gene	qRT-PCR	M + 25	*5’-AGA TGA GTC TTC TAA CCG AGG TCG-3’*	Spackman et al., 2002. [45]	Tissue and swab ^b^
			M 124	*5’-TGC AAA AAC ATC TTC TTC AAG TCT CTG-3’*		
			M + 64	*5’-FAM-TCA GGC CCC CTC AAA GCC GA-TAMRA-3’*		
**Rabies virus.**	Nucleoprotein	RT–PCR	RAB504	*5’-TAT ACT CGA ATC ATG AAT GGA GGT CGA CT-3’*	Primers: Oliveira et al. 2010. [46] Protocol: Carnieli et al. 2008 [47]	Tissue^c^
			RAB304	*5’-ACG CTT AAC AAC AAR ATC ARA G-3’*		
**Newcastle virus.**	Fusion gene, F0	RT-PCR	NCD3	*5’-GTC AAC ATA TAC ACC TCA TC-3’*	STAUBER, 1995. [48]	Tissue and swab ^b^
			NCD4	*5’-GGA GGA TGT TGG CAG CAT T-3’*		
***Toxoplasma gondii*.**	529bp repetitive segment	PCR	Tox-8	*5’-CCC AGC TGC GTC TGT CGG GAT-3’*	Homan et al., 2000. [49] Reischl et al., 2003. [50]	FFPE^d^
			Tox-11	*5’-GCG TCG TCT CGT CTA GAT CG-3’*		
***Trypanosoma cruzi*.**	18S rRNA gene	Nested PCR	First round: SSU4_F	*5’-GTG CCA GCA CCC GCG GTA AT-3’*	First round primer: Pinto et al., 2015. [51] Second round primer: Noyes et al., 1999. [52] Protocol: Aleman et al., 2017. [53] Murphy & O’Brien, 2007.[54]	FFPE^e^
			18Sq1R	*5’-CCA CCG ACC AAA AGC GGC CA-3’*		
			Second round: SSU561F	*5’-TGG GAT AAC AAA GGA GCA-3’*		
			SSU561R	*5’-CTG AGA CTG TAA CCT CAA AGC-3’*		
***Leishmania* spp.**	Kinetoplast	PCR	13A	*5’- GTG GGG GAG GGG CGT TCT-3’*	Medeiros et al. 2002. [55] Sosa-Ochoa et al. 2015. [56]	FFPE^f^
			13B	*5’-ATT TTA CAC CAA CCC CCA GTT-3’*		

FFPE: Formalin-fixed paraffin-embedded.

^a^ brain and lung.

^b^ Lung and Trachea tissue and cloacal swab.

^c^ hippocampus, cerebellum, and medulla oblongata.

^d^ spleen, lung, and liver.

^e^ heart.

^f^ spleen.

#### Detection of protozoan parasites`

Confirmation was performed using molecular techniques for pathogen identification when a previous presumptive protozoa presence was established in the histopathological study. All molecular methods were done in the presence of positive and negative controls. Tissue samples previously embedded in paraffin were used for this purpose. The deparaffinization procedure was done using xylol washes following the method recommended to perform DNA extraction from the tissue [57]. We performed DNA extraction using the commercial kit DNeasy Blood and Tissue (QIAGEN, Venlo, The Netherlands) according to the manufacturer’s instructions. The methods used and the samples collected are specified in Table 1.

#### Bacteriological detection

Tissue samples from animals with inflammatory processes (suppurative or abscesses) were cultured following standard bacteriological procedures. For bacterial isolation, samples were inoculated on non-selective and selective agar media. Significant bacterial growth was identified using the automated VITEK-2 Compact system, software version 8.02 (bioMérieux, Marcy l’Etoile, France). VITEK test cards for Gram-negative [GN], Gram-positive [GP], and anaerobes [ANC] were used for identification according to the manufacturer’s instructions.

#### Identification of metazoan parasites

All the parasites in the carcasses were collected and washed with physiological saline, preserved in alcohol, acetic acid, and formalin (AFA) solution. No more than one week after collection, they underwent identification to the genus level through morphometric characteristics [58]. Physical and morphometric characteristics were recognized after fixation and clarification with Hoyer’s solution by light microscopy [59–61]. In addition, processed cestodes were stained with dilute Harris’ hematoxylin solution.

### Information management, geocoding, and spatial analysis

The information on each case was included in the epidemiological surveillance information system (SIVE) from the animal health authority. Each case was geocoded using the latitude and longitude generated by GPS of the point where the specimen was found by field personnel. When the GPS was unavailable, they were geocoded using the latitude and longitude of the approximate location where they were found, and this was generated by Google Earth Pro v7.3 (2021, Google Inc.). With the georeferenced points of each sample admitted created a map using ArcGIS 10.7 (ERSI), according to territorial division by conservation area: Arenal Huetar Norte Conservation Area (ACAHN); Arenal Tempisque Conservation Area (ACT); Central Conservation Area (ACC); Guanacaste Conservation Area (ACG); La Amistad Caribe Conservation Area (ACLAC); La Amistad Pacífico Conservation Area (ACLAP); Osa Conservation Area (ACOSA); Pacífico Central Conservation Area (ACOPAC); Tempisque Conservation Area (ACT); Tortuguero Conservation Area (ACTo). Additionally, a feedback report was sent to the field staff with the relevant findings per case.

## Results

### Participation in the WHMP and distribution of cases by age, sex, and taxonomic classification

**Fig 2:**
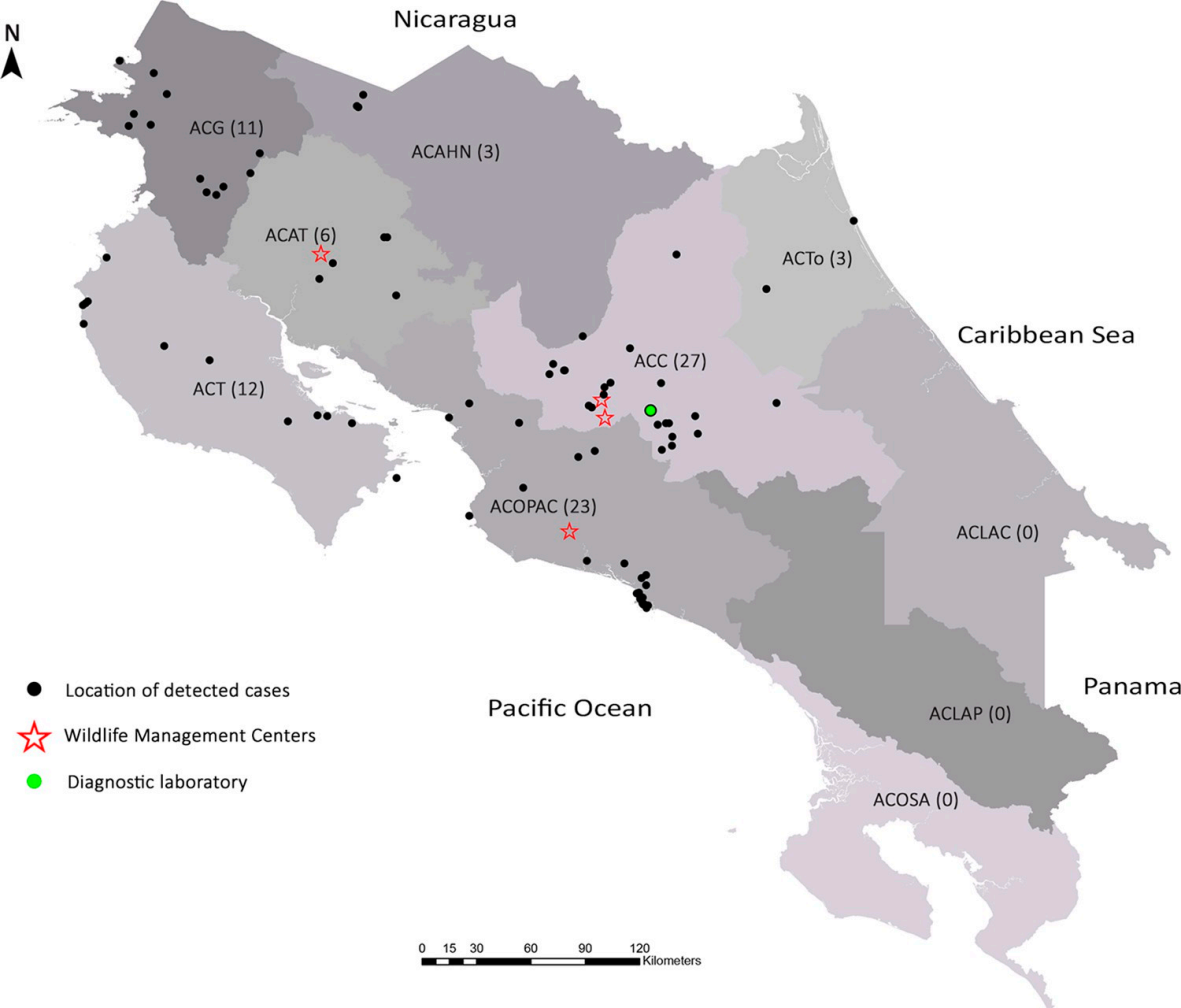
Geocoding of the cases analyzed by conservation area. The number corresponds to the cases analyzed in each conservation area. Wildlife management centers shown are those that collaborated with the WHMP.

The notification of cases was made by officials from the wildlife authority, with 24.7% (21/85) of the cases and 75.3% (64/85) by officials from wildlife management centers. Only four management centers reported and sent cases for analysis. The conservation areas with the most significant participation in the WHMP were the same ones where the participating wildlife management centers were located. The geographical location of the management centers, diagnostic laboratory, and cases analyzed is shown in Fig 2. The conservation areas where there was no participation are those located furthest from the diagnostic laboratory and with significant obstacles for shipment, as mentioned in Table 2.

**Table 2:** Participation in the WHMP of detectors of cases and obstacles found in each conservation area.

Conservation area	Number of cases	Cases detector		Obstacles to sending cases
		Wildlife Officer	MC	
ACAHN	3	0	3	Inability to store. Coordination problems with the health agency for the transport of specimens. Few rescue centers motivated to participate.
ACAT	6	0	6	Coordination problems with the wildlife agency to submit specimens.
ACC	27	6	21	No significant obstacles.
ACG	11	7	4	Coordination problems with the health agency for the transport of specimens.
ACLAC	0	0	0	Few rescue centers motivated to participate. Coordination problems with the wildlife agency to submit specimens. Coordination problems with the health agency for the transport of specimens. Distant from the diagnostic laboratory.
ACLAP	0	0	0	There are no rescue centers in the region. Coordination problems with the wildlife agency to submit specimens.
ACOSA	0	0	0	There are no rescue centers in the region. Distant from the diagnostic laboratory.
ACOPAC	23	0	23	Coordination problems with the wildlife agency to submit specimens.
ACT	12	5	7	Few rescue centers in the region. Inability to store
ACTo	3	3	0	There are no rescue centers in the region. Insufficient field staff. Coordination problems with the health agency for the transport of specimens.

ACAHN: Conservation area Arenal Huetar Norte; ACT: Conservation area Arenal Tempisque; ACC: Conservation area Central; ACG: Conservation area Guanacaste; ACLAC: Conservation area La Amistad Caribe; ACLAP: Conservation area La Amistad Pacifico; ACOSA: Conservation area Osa; ACOPAC: Conservation area pacific central; ACT: Conservation area Tempisque; ACTo: Conservation area Tortuguero.

Of the 85 specimens admitted to the study, there was an age distribution of 27.1% (23/85) young animals and 72.9% (62/85) adults. The sex distribution was 56.5% (48/85) males and 43.5% (37/85) females. According to the taxonomic order, we received 29.4% (25/85) Carnivora, 29.4% (25/85) Primate, 12.9% (11/85) Pilosa, 5.9% (5/85) Didelphimorphia, 4.7% (4/85) Rodentia, 4.7% (4/85) Artiodactyla, 2.3% (2/85) Cingulate, 2.3% (2/85) Pelecaniformes, 2.3% (2/85) Accipitriformes, 2.3% (2/85) Anseriformes, 1.2% (1/85) Ciconiiformes, 1.2% (1/85) Piciformes and 1.2% (1/85) Coraciiformes. The geographical distribution of admitted cases by conservation area is shown in Fig 2.

### Identification of causes of death according to pathological findings

According to pathological findings, the distribution of the presumptive cause of death corresponded to 60% (51/85) of death not associated with an infectious agent. Of these, 54.1% (46/85) associated with traumatic events (mainly roadkill and electrocution), 2.4% (2/85) with a degenerative disease, and in 3.5% (3/85) of cases, death was presumptively associated with intoxication. Additionally, of individuals with a cause of traumatic death, 37.6% (32/85) concomitantly presented some infectious agent with or without an associated disease (24 with gastrointestinal and pulmonary metazoan parasites, three with bacteria, one with protozoa, and four with multiple microorganisms). In 27.1% (23/85) of cases, death was directly related to infectious agents, ten presented lesions associated with viruses, five with metazoan parasites, two with protozoan parasites, one with bacteria, and five presented lesions associated with multiple etiologies. In 12.9% (11/85) of cases, the cause of death was not determined. The absolute and relative values of the causes of death for each taxonomic group according to the presence of infectious agents are specified in Table 3.

**Table 3:** Absolute and relative values of the causes of death for each taxonomic group.

Cause of Death / Taxon	DAIA	DNAIA-PD	DNAIA-IAD	DNAIA	UD
**Mammals**					
Carnivora	40% (10/25)	28% (7/25)	16% (4/25)	8% (2/25)	8% (2/25)
Primate	32% (8/25)	36% (9/25)	16% (4/25)	8% (2/25)	8% (2/25)
Pilosa	0% (0/11)	9.1% (1/11)	27.3% (3/11)	45.4% (5/11)	18.2% (2/11)
Didelphimorphia	20% (1/5)	0% (0/5)	60% (3/5)	0% (0/5)	20% (1/5)
Rodentia	25% (1/4)	0% (0/4)	0% (0/4)	25% (1/4)	50% (2/4)
Artiodactyla	25% (1/4)	0% (0/4)	0% (0/4)	75% (3/4)	0% (0/4)
Cingulate	0% (0/2)	0%% (0/2)	0%% (0/2)	100% (2/2)	0%% (0/2)
**Birds**					
Pelecaniformes	100% (2/2)	0% (2/2)	0% (2/2)	0% (2/2)	0% (2/2)
Accipitriformes	0% (0/2)	0% (0/2)	50% (1/2)	50% (1/2)	0% (0/2)
Anseriformes	0% (0/2)	0% (0/2)	0% (0/2)	100% (2/2)	0% (0/2)
Ciconiiformes	0% (0/1)	0% (0/1)	0% (0/1)	0% (0/1)	100% (1/1)
Piciformes	0% (0/1)	0% (0/1)	0% (0/1)	100% (1/1)	0% (0/1)
Coraciiformes	0% (0/1)	0% (0/1)	0% (0/1)	0% (0/1)	100% (1/1)
**Total**	27.1% (23/85)	20% (17/85)	17.6% (15/85)	22.4% (19/85)	12.9% (11/85)

DAIA: Death associated with an infectious agent; DNAIA-PD: Death not associated with an infectious agent, with a pre-existing infectious disease; DNAIA-IAD: Death not associated with an infectious agent, with infectious agent detection; DNAIA: Death not associated with an infectious agent; UD: Undetermined death.

### Infectious agents detected in the WHMP

**Fig 3:**
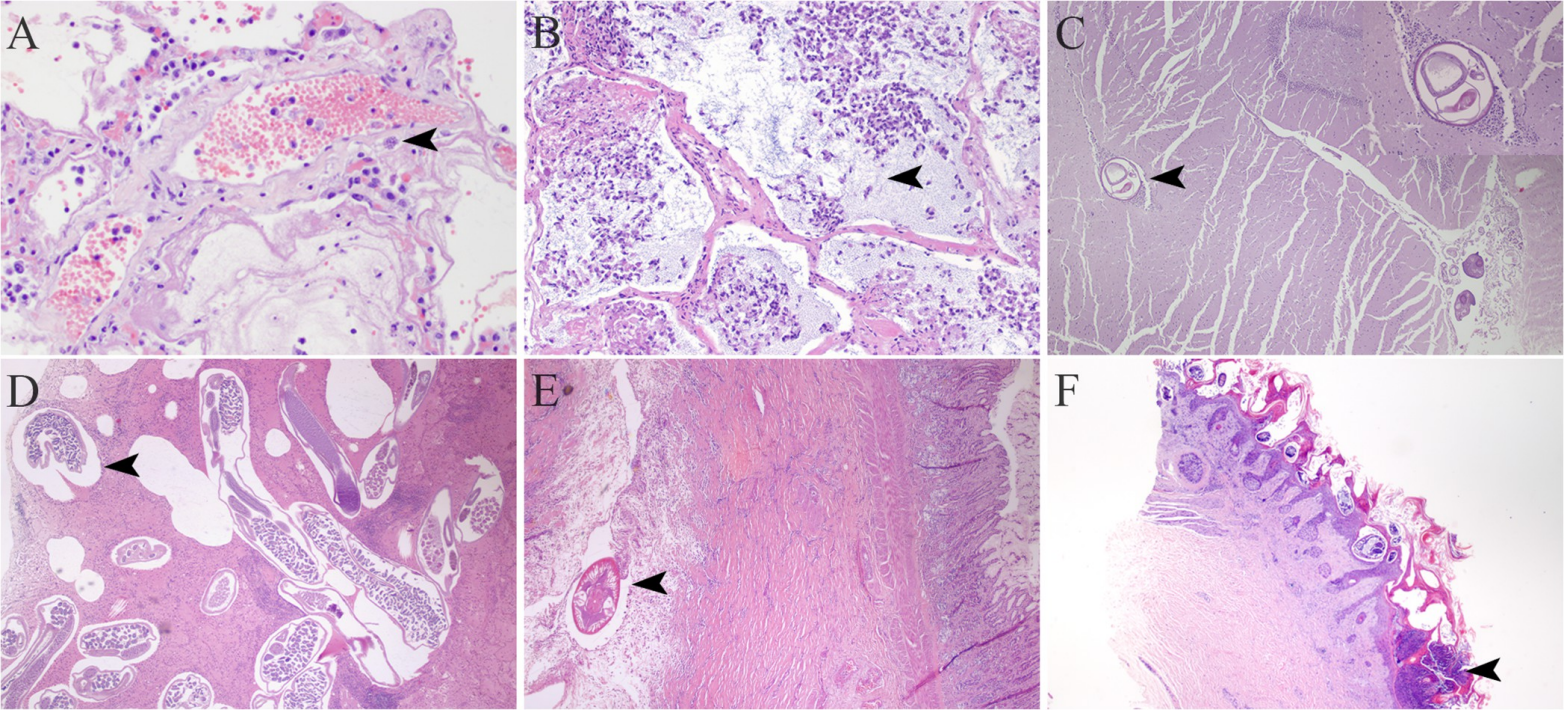
Infectious agents in lesions identified in wild animals. A) Lung (*Alouatta palliata*-howler monkey). Lymphoplasmacytic pneumonia with the presence of tissue cyst, morphology compatible with *Toxoplasma gondii*, confirmation by PCR (arrowhead; H&E 600x). B) Lung (*Alouatta palliata*-howler monkey). Pyogranulomatous abscessing bronchopneumonia with intralesional bacteria *Klebsiella pneumonia*, confirmation by culture (arrowhead; H&E 200x). C) Brain (*Didelphis marsupialis*-opossum). Presence of nematode *Angiostrongylus* spp. identified by morphology (arrowhead; H&E 400x). Inset: Nematode magnification (H&E 200x). D) Lung (*Cebus imitator*-white-faced monkey). Bronchopneumonia associated to multiple Nematodes, *Filariopsis* spp. identified by morphology (more cuts of the female in microphotograph) (arrowhead; H&E 40x). E) Stomach (*Herpailurus yagouaroundi*-jaguarundi). Nodular and sclerosing gastritis associated with multiple *Cylicospirura* spp. Nematodes identified by morphology (arrowhead; H&E 40x). F) Skin (*Sphiggurus mexicanus*-porcupine) Pyogranulomatous and eosinophilic dermatitis associated with massive infestation of *Sarcoptex* spp. (arrowhead; H&E 400x). Inset: Mites magnification (H&E 100x).

Ten viruses, seven protozoa, and seven bacteria were identified in mammalian specimens. In 22 cases, these pathogens were involved with lesions or systemic disease, of which 19 were directly associated with the cause of death of mammals. Only *Sarcocystis* spp. detected in two cases was an incidental finding. Additionally, 38 mammals had metazoan parasites. Multi-parasitosis was observed in 15.3% (13/85) of the cases. Parasites such as *Prosthenorchis* spp. (n = 15), *Angiostrongylus* spp. (n = 6), and *Cilycospirura* spp. (n = 1) were responsible for severe parasitosis with systemic disease. Some of the lesions, such as pyogranulomatous abscessing bronchopneumonia and nodular and sclerosing gastritis associated with infectious agents, are observed in Fig 3 (see legend). In 50.6% (43/85) of the cases, the mammals presented infectious agents with a zoonotic potential, such as *Klebsiella pneumoniae*, *Toxoplasma gondi*i, *Angiostrongylus* spp. The etiological agents identified by taxonomic groups and the number of specimens analyzed are specified in Table 4.

**Table 4:** Number of infectious agents tested and positive in mammals according to etiology.

Mammalian taxonomic groups / infectious agent		Primate	Carnivora	Pilosa	Didelphimorphia	Rodentia	Artiodactyla	Cingulate
Viral	CDV (n = 18)	0	10	0	0	0	0	0
	Alphaviruses (n = 9)	0	0	0	0	0	0	0
	Flaviviruses (n = 9)	0	0	0	0	0	0	0
	Influenza virus (n = 8)	0	0	0	0	0	0	0
	Rabies virus (n = 76)	0	0	0	0	0	0	0
Bacterial	*C*. *perfringens* (n = 18)	0	0	0	0	0	1	0
	*E*. *coli* (n = 18)	1	0	0	0	0	0	0
	*K*. *pneumoniae* (n = 18)	1	0	0	0	0	0	0
	*T*. *pyogenes*. (n = 18)	0	0	0	0	1	0	0
	*S*. *aureus* (n = 18)	1	1	1	0	0	0	0
	*Mycobacterium* spp. (n = 18)	0	0	0	0	0	0	0
Protozoan parasites	*Toxoplasma gondii* (n = 4)	2	0	0	0	0	0	0
	*Trypanosoma* spp. (n = 14)	0	0	0	3	0	0	0
	*Leishmania* spp. (n = 8)	0	0	0	0	0	0	0
	*Sarcocystis* spp. (n = 5)	0	1	0	0	0	1	0
**Metazoan parasites** ^**1**^	*Angiostrongylus* spp.	0	5	0	1	0	0	0
	*Dirofilaria* spp.	0	4	0	0	0	0	0
	*Dipetalonema* spp.	5	0	2	0	0	0	0
	*Gnathostoma* spp.	0	0	0	1	0	0	0
	*Baylisascaris* spp.	0	1	0	0	0	0	0
	*Ancylostoma* spp.	0	1	0	0	0	0	0
	*Cylicospirura* spp.	0	1	0	0	0	0	0
	*Prosthenorchis* spp.	10	5	0	0	0	0	0
	*Macracanthorhynchus* spp.	0	1	0	0	0	0	0
	*Spirometra* spp.	0	2	0	0	0	0	0

n: Number tested.

^1^ only zoonotic metazoan parasites are shown.

All birds submitted were evaluated for virus presence (n = 9); two of these were positive for flaviviruses. Additionally, three birds had metazoan parasites. Most of the pathogens identified were directly associated with the cause of the death of birds. Only *Procyrnea* spp. identified in one case was an incidental finding. In 2.3% (2/85) of the cases, the birds presented infectious agents with zoonotic potential, such as *Contracaecum* spp. The etiological agents identified in birds and the number of samples analyzed are specified in Table 5.

**Table 5:** Number of infectious agents tested and positive in birds according to etiology.

Avian taxonomic groups / infectious agent		Pelecaniformes	Accipitriformes	Anseriformes	Ciconiiformes	Piciformes	Coraciiformes
**Viral**	Alphaviruses (n = 3)	0	0	0	0	0	0
	Flaviviruses (n = 3)	2	0	0	0	0	0
	Influenza virus (n = 9)	0	0	0	0	0	0
	Newcastle virus (n = 9)	0	0	0	0	0	0
**Bacterial**	*C*. *perfringens* (n = 1)	0	0	0	0	0	0
	*E*. *coli* (n = 1)	0	0	0	0	0	0
	*K*. *pneumoniae* (n = 1)	0	0	0	0	0	0
	*Salmonella* spp. (n = 1)	0	0	0	0	0	0
	*S*. *aureus* (n = 1)	0	0	0	0	0	0
**Metazoan parasites** ^**1**^	*Contracaecum* spp.	2	0	0	0	0	0

n: Number of tested.

^1^ only zoonotic metazoan parasites are shown.

### Geospatial distribution of detected infectious agents and their accumulation by geographic region

**Fig 4:**
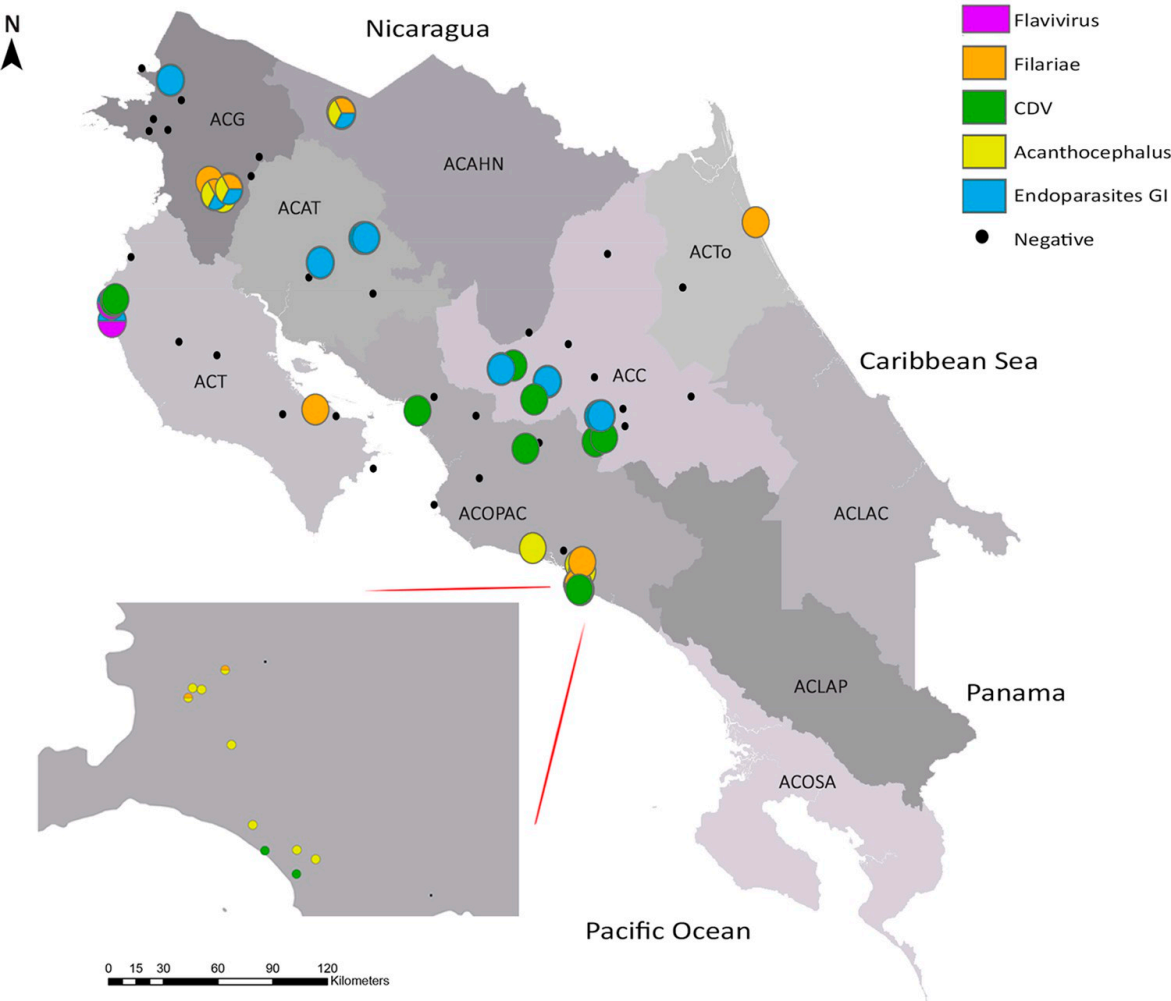
Geographical distribution of the most frequently identified infectious agents in the referred specimens. The individuals reported as negative were depicted even though the infectious agent was not detected in the complementary analyzes or no lesions suggestive of the disease were found in the pathological analysis.

We established the distribution of the most frequently identified infectious agents in the analyzed specimens (Fig 4). First, a wide distribution of zoonotic parasites was evidenced in the country. Then, there was an accumulation in the Central Pacific region of specimens with acanthocephaliasis (12 with *Prosthenorchis* spp., one with *Macracanthorhynchus* spp.), and an accumulation of specimens with gastrointestinal nematodes in the great metropolitan area and tourist areas of Guanacaste (six with *Angiostrongylus* spp., one with *Baylisascaris* spp., one with *Ancylostoma* spp.). Additionally, vector-borne diseases occurred exclusively in specimens from coastal regions and altitudes less than 300 meters above sea level (11 with filariae, two with flaviviruses). The CDV in carnivores from various areas of the country did not show a specific distribution pattern (n = 10). The analyzed specimens associated with these infectious agents can be observed in S1 Table.

## Discussion

The WHMP schemes have proven to be a fundamental tool in monitoring pathogens of zoonotic importance [62–64]. These surveillance systems are even more critical in geographical areas where high rates of biodiversity are prominent [14]. For example, Costa Rica is economically dependent on its ecotourism services, and its fauna is one of its most important assets [65]. However, no epidemiological surveillance system is currently directed to wildlife to study outbreaks or other health events.

Furthermore, implementing these types of schemes is essential for a country considered a "hotspot" for the appearance or emergence of new infectious agents; however, we encountered some obstacles when performing this study [11,13]. These obstacles are mainly related to lack of legislation for data collection, willingness to cooperate between agencies, financial disincentives and logistical problems for the storage and transport of carcasses, and difficulties similar to those described by some authors [66,67].

The storage capacity and transport logistics directly impact WHMPs. Urban areas with transportation facilities reported and dispatched more carcasses, in contrast to remote or difficult-to-access regions with less participation. These patterns tally with previous reports indicating that notification of wildlife mortality or morbidity generally depends on the initial detection of cases by the general public. Consequently, detected cases are biased towards events in populated or easily accessible areas [17,68,69]. Nevertheless, this shows that the existing logistics in ACC and ACOPAC (urban areas with the highest number of reported cases) can be used to maintain the WHMP at least on a regional level. Likewise, it is necessary to expand the network of laboratories to include other institutions with pathological diagnosis capacity within the WHMP scheme to reduce reliance on the storage and transportation of carcasses. This measure has been shown to improve coverage in distant regions and increase case reporting [19,20,63].

The lack of guidelines and legislation also limited participation and case detection. This means that the system is maintained by the self-interest of officials and interpersonal relationships of people from different institutions. These findings are consistent with previous evaluations of the veterinary services of Costa Rica [26]. To ensure the long-term sustainability of the WHMP, legislation and regulations are necessary to provide financial support and clarify the specific functions of each institution. This could facilitate coordination and cooperation between institutions to notify and transport specimens [19,20,62,63].

The notification and referral of cases relied heavily on the management centers that provide veterinary care to wild animals. Other studies have proposed these institutions as an indispensable tool within the WHMPs due to the large amount of information they can generate for the system [70,71]. The performance of necropsy by the veterinary doctors of management centers would greatly support the efficiency and sustainability of this WHMP, reducing the demand for transportation (only samples would be transported, not complete carcasses), and it would eliminate freezing; facilitating diagnosis. This could encourage the participation of management centers from distant regions without storage capacity, thus increasing coverage. However, the previous regulations, manuals and procedures for post-mortem analysis and sampling procedures are necessary, as occurs with other surveillance schemes, to avoid affecting the diagnosis since pathological analysis is vital in passive surveillance schemes [17,20,21].

Carnivores and primates were the taxa with higher representation. These data can be associated with the fact that they are medium to large-sized animals, more charismatic, and with a more significant contact of these species with human environments, facilitating the recognition of morbidities and mortalities by the population [69]. Therefore, taxa should be prioritized in the WHMPs, since it allows for optimizing the use of resources. Furthermore, in addition to their easy detection, they are taxa in which various pathogenic agents can circulate [5,7].

In contrast, obtaining viable bird carcasses for post-mortem analysis was challenging due to the advanced degree of autolysis, wasting important transport and storage resources, an obstacle experienced in other studies [72]. Wild birds should be included in surveillance programs for influenza virus and Newcastle disease virus that maintains in the poultry production systems in the country. This would maintain monitoring as is done in other WHMPs without the need for post-mortem analysis, thus avoiding wasted resources [19].

In addition, most cases with a traumatic cause of death presented some pre-existing infectious pathology. Free-living animals are naturally exposed to infectious agents, so it is common to find them in incidental lesions in post-mortem analysis [73–75]. These cases allow the detection of infectious agents in passive surveillance, whether or not they are associated with the cause of death [73,76–78]. Therefore, they must be kept within the cases to be analyzed.

The diagnostic capability allowed the WHMP to detect infectious agents that could affect the health of domestic animals, public health, and the conservation of wild species. For example, our study shows the presence of potentially zoonotic bacterial infectious agents classified as emerging diseases in some regions [79–81]. The most relevant are *Klebsiella pneumoniae*, *Escherichia coli*, and *Staphylococcus aureus*, which were associated with primary disease in some of the analyzed specimens. In addition, these bacteria currently top the list of infectious agents with antibiotic resistance genes, thus showing the importance of monitoring these agents in WHMP schemes [28,82–84].

We also detect vector-borne diseases, which are recognized as agents with epidemic potential in Latin America due to tropical regions’ climatic, health, and socioeconomic conditions that favor their spread [85–87]. We identified primates, carnivores, and birds with infectious agents of vector transmission, for example, *Dirofilaria* spp., *Dipetalonema* spp., and flavivirus mainly present by the coast. Most of these cases come from regions already defined as endemic areas for these infectious agents in domestic animals, which reveals a possible transmission by this route and a potential risk for the conservation of the species [88–91].

Detection of these vector-borne pathogens also reveals a potential risk to public health in places with a high rate of tourists visiting Costa Rica. This risk is reinforced by health system reports showing at least three disease cases in humans associated with *Dirofilaria immitis* and isolated cases of subcutaneous filariasis [92–94]. Furthermore, detecting virus-related mortalities such as West Nile in wild birds (as was possibly our case) allows early alerts. It has been shown that there is a higher risk of exposure for human populations close to the regions where mortalities of wild birds occur [95,96].

The CDV was frequently detected in our study, reflecting the relevance of this virus in the role of spillover towards carnivore species and possibly the implications of a spillback towards susceptible or non-vaccinated domestic canines [97–99]. Endemic CDV outbreaks have been reported anecdotally throughout Costa Rica and America in dog populations. More recently, sporadic outbreaks in wild carnivores of urban and suburban areas have been recorded [97,100]. Unfortunately, Costa Rica does not have official data on the domestic dog population. Therefore, herd immunity data in this population is uncertain, especially for dogs without an owner or in non-urban areas. This poses a risk to wild carnivores, especially in urban areas with susceptible canine populations. Furthermore, the possibility of transmission of this virus to other species beyond carnivores is a hypothesis that has been investigated [101]. Given the high diversity of vertebrates in Costa Rica and the high circulation of CDV detected, this virus should be considered within epidemiological surveillance programs.

Also, this study’s gastrointestinal and pulmonary metazoan parasites are relevant for public health and wildlife conservation programs. For instance, we detect the nematodes *Angiostrongylus* spp., *Baylisascaris* spp., *Ancylostoma* spp., and *Cylicospirura* spp. in mammalian species located in densely populated areas. In addition, we detected cases with acanthocephalans (*Prosthenorchis* spp., *Macracanthorhynchus* spp.) concentrated in the Central Pacific region and parasites transmitted by water or aquatic food such as the cestode *Spirometra* spp. in the country’s northern region. This result proves a cost-effective tool for the WHMP, which does not require financial resources beyond qualified personnel for the morphological identification of worms and allows the detection of pathogenic agents that primarily impact children [102–104].

This study did not detect rabies virus infections. These findings are supported by previous studies on wild animals in Costa Rica [29]. However, this passive surveillance program allowed expanding coverage in the number of species and geographic regions through constant monitoring of wild species. Human and livestock fatalities have been reported associated with rabies infections, which stresses the relevance of its continuous monitoring of species that can act as reservoirs [35,105]. A similar situation applies to Newcastle and Influenza virus. In our samples, none of the birds showed evidence of disease or associated clinical signs; however, due to the great relevance of these diseases to the country and the risk for national production, it is advisable to establish routine monitoring by the animal health agency under the WHMP scheme [35,106]. Including serological monitoring of tuberculosis and brucellosis of wild species in the scheme would even be advisable. These national epidemiological surveillance programs already include some wild species, and coverage could be expanded [107].

Finally, we could not identify the cause of death in some of the samples analyzed. Although we tested for the main circulating infectious agents in Costa Rica, no conclusive data was obtained. Ranges of 17–22% have been reported in pathological studies in wild species, where the causative agent of the disease cannot be determined, mainly associated with the degree of autolysis and the diagnostic complexity [68,74,76]. These results are consistent with the percentages of an absence of identification of the etiological agent in our samples. Although proving that diagnostic capacity is acceptable, further work is necessary to develop robust diagnostic techniques for wild animal testing. Further efforts and incentives, financed by government authorities, are required for pathogen surveillance in wildlife through the consistent implementation of tools such as new generation metagenomics [108–111].

Although the proposed program is limited to the country’s resources and infrastructure, and it is clear that it is not generally applicable, it is important to start evaluating the implementation of these programs in regions where disease surveillance in wildlife is minimal. For example, this study shows that this passive surveillance scheme is cost-effective and feasible to establish in countries with limited resources. Furthermore, this scheme was possible since we could adapt the infrastructure dedicated to monitoring diseases in productive animals according to the scope and objectives of monitoring wildlife specific to each region. We also showed sufficient diagnostic capacity in the country for detecting infectious agents of zoonotic and conservation importance in wild animals. If this scheme is maintained over time, it will generate data to allow the decision-making to promote the conservation of species, animal health, and public health by knowing the circulation and behavior of these pathogens [68].

This study highlights the need for an inter-institutional and trans-institutional commitment to the sustainability over time of this surveillance scheme. Participant institutions must remain motivated and focused on the benefits beyond the economic part. The feedback to field staff and the frequent reports of the importance of detected pathogens are crucial to maintaining motivation and detection network, as in our case. In addition, the information generated from the experience of the initial establishment of a WHMP is critical to meeting the challenges involved in developing this type of scheme in regions with limited resources and established as hotspots for emerging infectious diseases [13,112]. Although it is necessary to standardize methods and techniques for monitoring pathogens in wildlife, the development of pilot schemes allows sharing experiences with programs already installed and leads to subsequent optimization and standardization studies that will facilitate the exchange of information and expand coverage [112].

## Supporting information

S1 Table Biological data and more representative pathological findings of the analyzed specimens. (DOCX)

## References

[1] Morens DM, Folkers GK, Fauci AS. The challenge of emerging and re-emerging infectious diseases. Nature. 2004; 430:242–9. doi: 10.1038/nature02759 . 15241422 PMC7094993

[2] Bloom DE, Cadarette D. Infectious Disease Threats in the Twenty-First Century: Strengthening the Global Response. Front Immunol. 2019; 10:549. doi: 10.3389/fimmu.2019.00549 . 30984169 PMC6447676

[3] Bakonyi T, Haussig JM. West Nile virus keeps on moving up in Europe. Euro Surveill. 2020; 25. doi: 10.2807/1560-7917.ES.2020.25.46.2001938 . 33213684 PMC7678036

[4] Barrett ADT. The reemergence of yellow fever. Science. 2018; 361:847–8. Epub 2018/08/23. doi: 10.1126/science.aau8225 . 30139914

[5] Han BA, Kramer AM, Drake JM. Global Patterns of Zoonotic Disease in Mammals. Trends Parasitol. 2016; 32:565–77. Epub 2016/06/14. doi: 10.1016/j.pt.2016.04.007 . 27316904 PMC4921293

[6] Hassell JM, Begon M, Ward MJ, Fèvre EM. Urbanization and Disease Emergence: Dynamics at the Wildlife-Livestock-Human Interface. Trends Ecol Evol (Amst). 2017; 32:55–67. doi: 10.1016/j.tree.2016.09.012 . 28029378 PMC5214842

[7] Plourde BT, Burgess TL, Eskew EA, Roth TM, Stephenson N, Foley JE. Are disease reservoirs special? Taxonomic and life history characteristics. PLoS One. 2017; 12:e0180716. Epub 2017/07/13. doi: 10.1371/journal.pone.0180716 . 28704402 PMC5509157

[8] Reif JS. Animal sentinels for environmental and public health. Public Health Rep. 2011; 126 Suppl 1:50–7. doi: 10.1177/00333549111260S108 . 21563712 PMC3072903

[9] Kowalewski MM, Salzer JS, Deutsch JC, Raño M, Kuhlenschmidt MS, Gillespie TR. Black and gold howler monkeys (Alouatta caraya) as sentinels of ecosystem health: patterns of zoonotic protozoa infection relative to degree of human-primate contact. Am J Primatol. 2011; 73:75–83. doi: 10.1002/ajp.20803 . 20084672

[10] Jones KE, Patel NG, Levy MA, Storeygard A, Balk D, Gittleman JL, et al. Global trends in emerging infectious diseases. Nature. 2008; 451:990–3. doi: 10.1038/nature06536 . 18288193 PMC5960580

[11] Walsh MG, Sawleshwarkar S, Hossain S, Mor SM. Whence the next pandemic? The intersecting global geography of the animal-human interface, poor health systems and air transit centrality reveals conduits for high-impact spillover. One Health. 2020; 11:100177. Epub 2020/10/08. doi: 10.1016/j.onehlt.2020.100177 . 33052311 PMC7543735

[12] White RJ, Razgour O. Emerging zoonotic diseases originating in mammals: a systematic review of effects of anthropogenic land-use change. Mamm Rev. 2020. Epub 2020/06/02. doi: 10.1111/mam.12201 . 32836691 PMC7300897

[13] Allen T, Murray KA, Zambrana-Torrelio C, Morse SS, Rondinini C, Di Marco M, et al. Global hotspots and correlates of emerging zoonotic diseases. Nat Commun. 2017; 8:1124. Epub 2017/10/24. doi: 10.1038/s41467-017-00923-8 . 29066781 PMC5654761

[14] Petrovan SO, Aldridge DC, Bartlett H, Bladon AJ, Booth H, Broad S, et al. Post COVID-19: a solution scan of options for preventing future zoonotic epidemics. Biol Rev Camb Philos Soc. 2021. Epub 2021/07/07. doi: 10.1111/brv.12774 . 34231315 PMC8444924

[15] World Health Organization. Anticipating Emerging Infectious Disease Epidemics. Switzerland: WHO 2015. Available from: https://apps.who.int/iris/bitstream/handle/10665/252646/WHO-OHE-PED-2016.2-eng.pdf.

[16] World Organisation for Animal Health. Training manual on surveillance and international reporting of diseases in wild animals second cycle. Workshop for OIE National Focal Points for Wildlife. Paris: OIE 2015. Available from: https://www.oie.int/fileadmin/Home/eng/Internationa_Standard_Setting/docs/pdf/WGWildlife/A_Training_Manual_Wildlife_2.pdf.

[17] Sleeman JM. Strategies for Wildlife Disease Surveillance. United States: U.S. Geological Survey National Wildlife Health Center 2012. Available from: https://digitalcommons.unl.edu/cgi/viewcontent.cgi?article=1981&context=usgsstaffpub.

[18] Guberti V, Stancampiano L, Ferrari N. Surveillance, monitoring and survey of wildlife diseases: a public health and conservation approach. Hystrix, the Italian Journal of Mammalogy. 2014; 25. doi: 10.4404/hystrix-25.1–10114

[19] Lamarque F. Le reseau sagir, reseau national de suivi sanitaire de la faune sauvage française. Paris: Epidémiol. et santé anim 2000. Available from: https://www.researchgate.net/profile/Marc_Artois/publication/237744255_Le_reseau_SAGIR_reseau_national_de_suivi_sanitaire_de_la_faune_sauvage_francaise/links/00b7d529709d2110a0000000/Le-reseau-SAGIR-reseau-national-de-suivi-sanitaire-de-la-faune-sauvage-francaise.pdf.

[20] Leighton FA, Wobeser GA, Barker IK, Daoust PY, Martineau D. The Canadian Cooperative Wildlife Health Centre and surveillance of wild animal diseases in Canada. Can Vet J. 1997; 38:279–84. Available from: https://www.ncbi.nlm.nih.gov/pmc/articles/PMC1576906/pdf/canvetj00090-0025.pdf. 9167876 PMC1576906

[21] Guia de vigilância de epizootias em primatas não humanos e entomologia aplicada à vigilância da febre amarela. 2nd ed. Brasília D.F.: Ministério da Saúde, Secretaria de Vigilância em Saúde, Departamento de Vigilância das Doenças Transmissiveis; 2014. Available from: https://bvsms.saude.gov.br/bvs/publicacoes/guia_vigilancia_epizootias_primatas_entomologia.pdf.

[22] Servicio Nacional de Sanidad y Calidad Agroalimentaria (SENASA). Informe de Notificaciones de Enfermedades Denunciables. Trichinellosis. Argentina: Ministerio de Agricultura, Ganaderia y Pesca 2019. Available from: https://www.argentina.gob.ar/sites/default/files/informe_trichinellosis_2010_2019.pdf.

[23] Sanchez-Vazquez MJ, Hidalgo-Hermoso E, Zanette LC, Campos Binder L de, Rivera, Molina-Flores B, et al. Characteristics and Perspectives of Disease at the Wildlife-Livestock Interface in Central and South America. In: Vicente J, Vercauteren KC, Gortázar C, editors. Diseases at the Wildlife—Livestock Interface. Cham: Springer International Publishing; 2021. pp. 271–304. doi: 10.1007/978-3-030-65365-1_9

[24] Rojas H, Romero JR. Where to next with animal health in Latin America? The transition from endemic to disease-free status. Rev—Off Int Epizoot. 2017; 36:331–48. doi: 10.20506/rst.36.1.2633 . 28926004

[25] United States Department of Agriculture (USDA). Report on the Review of Costa Rica’s Animal Health Statuses. Estados Unidos: United States Department of Agriculture 2019. Available from: https://www.aphis.usda.gov/import_export/downloads/costarica-status-review.pdf.

[26] World Organisation for Animal Health. Informe de Misión Piloto Evaluación PVS “Una Salud”. Paris: OIE 2011. Available from: https://www.oie.int/fileadmin/Home/eng/Support_to_OIE_Members/pdf/InterimReport-Costa_Rica.pdf.

[27] Baldi M, Alvarado G, Smith S, Santoro M, Bolaños N, Jiménez C, et al. Baylisascaris procyonis Parasites in Raccoons, Costa Rica, 2014. Emerging Infect Dis. 2016; 22:1502–3. doi: 10.3201/eid2208.151627 . 27433741 PMC4982188

[28] Baldi M, Barquero Calvo E, Hutter SE, Walzer C. Salmonellosis detection and evidence of antibiotic resistance in an urban raccoon population in a highly populated area, Costa Rica. Zoonoses Public Health. 2019; 66:852–60. Epub 2019/07/29. doi: 10.1111/zph.12635 . 31359623 PMC6852039

[29] Baldi M, Hernández-Mora G, Jimenez C, Hutter SE, Alfaro A, Walzer C. Leptospira Seroprevalence Detection and Rabies Virus Absence in an Urban Raccoon (Procyon lotor) Population in a Highly Populated Area, Costa Rica. Vector Borne Zoonotic Dis. 2019; 19:889–95. Epub 2019/08/13. doi: 10.1089/vbz.2019.2444 . 31407956

[30] Chaves A, Piche-Ovares M, Ibarra-Cerdeña CN, Corrales-Aguilar E, Suzán G, Moreira-Soto A, et al. Serosurvey of Nonhuman Primates in Costa Rica at the Human-Wildlife Interface Reveals High Exposure to Flaviviruses. Insects. 2021; 12. Epub 2021/06/15. doi: 10.3390/insects12060554 . 34203687 PMC8232092

[31] Dolz G, Chaves A, Gutiérrez-Espeleta GA, Ortiz-Malavasi E, Bernal-Valle S, Herrero MV. Detection of antibodies against flavivirus over time in wild non-human primates from the lowlands of Costa Rica. PLoS One. 2019; 14:e0219271. doi: 10.1371/journal.pone.0219271 . 31276532 PMC6611622

[32] Dubey JP, Morales JA, Sundar N, Velmurugan GV, González-Barrientos CR, Hernández-Mora G, et al. Isolation and genetic characterization of Toxoplasma gondii from striped dolphin (Stenella coeruleoalba) from Costa Rica. J Parasitol. 2007; 93:710–1. doi: 10.1645/GE-1120R.1 . 17626370

[33] Fuentes-Ramírez A, Jiménez-Soto M, Castro R, Romero-Zuñiga JJ, Dolz G. Molecular Detection of Plasmodium malariae/Plasmodium brasilianum in Non-Human Primates in Captivity in Costa Rica. PLoS One. 2017; 12:e0170704. doi: 10.1371/journal.pone.0170704 . 28125696 PMC5268763

[34] González K, Calzada JE, Saldaña A, Rigg CA, Alvarado G, Rodríguez-Herrera B, et al. Survey of wild mammal hosts of cutaneous leishmaniasis parasites in Panamá and Costa Rica. Trop Med Health. 2015; 43:75–8. Epub 2014/12/06. doi: 10.2149/tmh.2014-30 . 25859156 PMC4361339

[35] Hutter SE, Brugger K, Sancho Vargas VH, González R, Aguilar O, León B, et al. Rabies in Costa Rica: Documentation of the Surveillance Program and the Endemic Situation from 1985 to 2014. Vector Borne Zoonotic Dis. 2016; 16:334–41. Epub 2016/03/16. doi: 10.1089/vbz.2015.1906 . 26982168 PMC4841904

[36] Patiño W LC, Monge O, Suzán G, Gutiérrez-Espeleta G, Chaves A. Molecular Detection of Mycobacterium avium avium and Mycobacterium genavense in Feces of Free-living Scarlet Macaws (Ara macao) in Costa Rica. J Wildl Dis. 2018; 54:357–61. doi: 10.7589/2017-05-124 . 29286261

[37] Pérez-Gómez G, Jiménez-Rocha AE, Bermúdez-Rojas T. Parásitos gastrointestinales de aves silvestres en un ecosistema ribereño urbano tropical en Heredia, Costa Rica. RBT. 2018; 66:788. doi: 10.15517/rbt.v66i2.33409

[38] Rojas-Jiménez J, Morales-Acuña JA, Argüello-Sáenz M, Acevedo-González SE, Yabsley MJ, Urbina-Villalobos A. Histopathological findings of infections caused by Canine Distemper virus, Trypanosoma cruzi, and other parasites in two free-ranging White-nosed Coatis Nasua narica (Carnivora: Procyonidae) from Costa Rica. J Threat Taxa. 2021; 13:17521–8. doi: 10.11609/jott.5907.13.1.17521–17528

[39] World Organisation for Animal Health. Training manual on wildlife diseases and surveillance. Workshop for OIE National Focal Points for Wildlife. Paris: OIE 2010. Available from: https://www.oie.int/fileadmin/Home/eng/Internationa_Standard_Setting/docs/pdf/WGWildlife/A_Training_Manual_Wildlife.pdf.

[40] McAloose D, Colegrove KM, Newton AL. Wildlife Necropsy. Pathology of Wildlife and Zoo Animals. Elsevier; 2018. pp. 1–20.

[41] Woodford MH, Bengis RG, Keet DF. Post-mortem procedures for wildlife veterinarians and field biologists. Paris: OIE; 2000.

[42] Da Budaszewski RF, Pinto LD, Weber MN, Caldart ET, Alves CDBT, Martella V, et al. Genotyping of Canine Distemper virus strains circulating in Brazil from 2008 to 2012. Virus Res. 2014; 180:76–83. Epub 2013/12/24. doi: 10.1016/j.virusres.2013.12.024 . 24370870

[43] Grywna K, Kupfer B, Panning M, Drexler JF, Emmerich P, Drosten C, et al. Detection of all species of the genus Alphavirus by reverse transcription-PCR with diagnostic sensitivity. J Clin Microbiol. 2010; 48:3386–7. doi: 10.1128/JCM.00317-10 . 20504990 PMC2937745

[44] Scaramozzino N, Crance JM, Jouan A, DeBriel DA, Stoll F, Garin D. Comparison of flavivirus universal primer pairs and development of a rapid, highly sensitive heminested reverse transcription-PCR assay for detection of flaviviruses targeted to a conserved region of the NS5 gene sequences. J Clin Microbiol. 2001; 39:1922–7. doi: 10.1128/JCM.39.5.1922-1927.2001 . 11326014 PMC88049

[45] Spackman E, Senne DA, Myers TJ, Bulaga LL, Garber LP, Perdue ML, et al. Development of a real-time reverse transcriptase PCR assay for type A influenza virus and the avian H5 and H7 hemagglutinin subtypes. J Clin Microbiol. 2002; 40:3256–60. doi: 10.1128/JCM.40.9.3256-3260.2002 . 12202562 PMC130722

[46] Oliveira RdN, Souza SP de, Lobo RSV, Castilho JG, Macedo CI, Carnieli P, et al. Rabies virus in insectivorous bats: implications of the diversity of the nucleoprotein and glycoprotein genes for molecular epidemiology. Virology. 2010; 405:352–60. doi: 10.1016/j.virol.2010.05.030 . 20609456

[47] Carnieli P, Fahl WdO, Castilho JG, Oliveira RdN, Macedo CI, Durymanova E, et al. Characterization of Rabies virus isolated from canids and identification of the main wild canid host in Northeastern Brazil. Virus Res. 2008; 131:33–46. Epub 2007/09/21. doi: 10.1016/j.virusres.2007.08.007 . 17889396

[48] Stauber N. Detection of Newcastle disease virus in poultry vaccines using the polymerase chain reaction and direct sequencing of amplified cDNA. Vaccine. 1995; 13:360–4. doi: 10.1016/0264-410x(95)98257-b 7793131

[49] Homan W, Vercammen M, Braekeleer J, Verschueren H. Identification of a 200- to 300-fold repetitive 529 bp DNA fragment in Toxoplasma gondii, and its use for diagnostic and quantitative PCR1Note: Nucleotide sequence data reported in this paper have been submitted to GenBankTM database with the accession number AF146527 (Toxoplasma gondii genomic repetitive 529 bp fragment).1. International Journal for Parasitology. 2000; 30:69–75. doi: 10.1016/s0020-7519(99)00170-8 10675747

[50] Reischl U, Bretagne S, Krüger D, Ernault P, Costa J-M. Comparison of two DNA targets for the diagnosis of Toxoplasmosis by real-time PCR using fluorescence resonance energy transfer hybridization probes. BMC Infect Dis. 2003; 3:7. doi: 10.1186/1471-2334-3-7 . 12729464 PMC156600

[51] Pinto CM, Ocaña-Mayorga S, Tapia EE, Lobos SE, Zurita AP, Aguirre-Villacís F, et al. Bats, Trypanosomes, and Triatomines in Ecuador: New Insights into the Diversity, Transmission, and Origins of Trypanosoma cruzi and Chagas Disease. PLoS One. 2015; 10:e0139999. Epub 2015/10/14. doi: 10.1371/journal.pone.0139999 . 26465748 PMC4605636

[52] Noyes H, Stevens J, Teixeira M, Phelan J, Holz P. A nested PCR for the ssrRNA gene detects *Trypanosoma binneyi* in the platypus and *Trypanosoma* sp. in wombats and kangaroos in Australia1. International Journal for Parasitology. 1999; 29:331–9. doi: 10.1016/S0020-7519(98)00167-2 10221634

[53] Aleman A, Guerra T, Maikis TJ, Milholland MT, Castro-Arellano I, Forstner MRJ, et al. The Prevalence of Trypanosoma cruzi, the Causal Agent of Chagas Disease, in Texas Rodent Populations. Ecohealth. 2017; 14:130–43. Epub 2017/01/13. doi: 10.1007/s10393-017-1205-5 . 28091763

[54] Murphy WJ O’Brien SJ. Designing and optimizing comparative anchor primers for comparative gene mapping and phylogenetic inference. Nat Protoc. 2007; 2:3022–30. doi: 10.1038/nprot.2007.429 . 18007639

[55] Medeiros ACR, Rodrigues SS, Roselino AMF. Comparison of the specificity of PCR and the histopathological detection of leishmania for the diagnosis of American cutaneous leishmaniasis. Braz J Med Biol Res. 2002; 35:421–4. doi: 10.1590/s0100-879x2002000400002 . 11960189

[56] Sosa-Ochoa W, Morales Cortedano X, Argüello S, Zuniga C, Henríquez J, Mejía R, et al. Ecoepidemiología de la Leishmaniasis cutánea no ulcerada en Honduras. Ciencia y Tecnología. 2015:115–28. doi: 10.5377/rct.v0i14.1799

[57] Körbler T, Gršković M, Dominis M, Antica M. A simple method for RNA isolation from formalin-fixed and paraffin-embedded lymphatic tissues. Experimental and Molecular Pathology. 2003; 74:336–40. doi: 10.1016/s0014-4800(03)00024-8 12782023

[58] Mehlhorn H. Methods to Diagnose Parasites. In: Mehlhorn H, editor. Animal Parasites. Cham: Springer International Publishing; 2016. pp. 23–32.

[59] Mehlhorn H. Worms (Helminths). In: Mehlhorn H, editor. Animal Parasites. Cham: Springer International Publishing; 2016. pp. 251–498.

[60] Mehlhorn H. Worms of Humans. In: Mehlhorn H, editor. Human Parasites. Cham: Springer International Publishing; 2016. pp. 135–298.

[61] Salfelder K, Liscano TR de, Sauerteig E. Helminthic diseases. In: Salfelder K, Liscano R de, Sauerteig E, editors. Atlas of Parasitic Pathology. Dordrecht: Springer Netherlands; 1992. pp. 96–172.

[62] Childs JE, Krebs JW, Real LA, Gordon ER. Animal-based national surveillance for zoonotic disease: quality, limitations, and implications of a model system for monitoring rabies. Prev Vet Med. 2007; 78:246–61. Epub 2006/11/28. doi: 10.1016/j.prevetmed.2006.10.014 . 17129622 PMC7114326

[63] Moede Rogall G, Sleeman JM. The USGS National Wildlife Health Center: Advancing wildlife and ecosystem health. Reston, VA; 2017. doi: 10.3133/fs20163102

[64] Stephen Craig. CWHC annual report 2019/2020. Canada: Canadia Wildlife Health Cooperative 2020. Available from: http://www.cwhc-rcsf.ca/docs/annual_reports/2019_2020_CWHC_Annual_Report_EN.pdf?v=20201207.

[65] Benavides Vindas S. El aporte del turismo a la economía costarricense: más de una década después. Econom y Socied. 2020; 25:1–29. doi: 10.15359/eys.25-57.1

[66] Stitt T, Mountifield J, Stephen C. Opportunities and obstacles to collecting wildlife disease data for public health purposes: results of a pilot study on Vancouver Island, British Columbia. Can Vet J. 2007; 48:83–7, 89–90. doi: 10.4141/cjas68-011 . 17310627 PMC1716737

[67] Don Bamunusinghage Nihal P, Dangolla A, Hettiarachchi R, Abeynayake P, Stephen C. Challenges and opportunities for wildlife disease surveillance in Sri Lanka. J Wildl Dis. 2020; 56:538–46. Epub 2020/01/09. doi: 10.7589/2019-07-181 . 31917632

[68] Pewsner M, Origgi FC, Frey J, Ryser-Degiorgis M-P. Assessing Fifty Years of General Health Surveillance of Roe Deer in Switzerland: A Retrospective Analysis of Necropsy Reports. PLoS One. 2017; 12:e0170338. Epub 1/19/2017. doi: 10.1371/journal.pone.0170338 . 28103325 PMC5245894

[69] Stallknecht DE. Impediments to wildlife disease surveillance, research, and diagnostics. Curr Top Microbiol Immunol. 2007; 315:445–61. doi: 10.1007/978-3-540-70962-6_17 . 17848074

[70] Cox-Witton K, Reiss A, Woods R, Grillo V, Baker RT, Blyde DJ, et al. Emerging infectious diseases in free-ranging wildlife-Australian zoo based wildlife hospitals contribute to national surveillance. PLoS One. 2014; 9:e95127. Epub 2014/05/01. doi: 10.1371/journal.pone.0095127 . 24787430 PMC4006786

[71] Gourlay P, Decors A, Moinet M, Lambert O, Lawson B, Beaudeau F, et al. The potential capacity of French wildlife rescue centres for wild bird disease surveillance. Eur J Wildl Res. 2014; 60:865–73. doi: 10.1007/s10344-014-0853-9

[72] Balseiro A, Espí A, Márquez I, Pérez V, Ferreras MC, Marín JFG, et al. Pathological features in marine birds affected by the prestige’s oil spill in the north of Spain. J Wildl Dis. 2005; 41:371–8. doi: 10.7589/0090-3558-41.2.371 . 16107672

[73] Pedro Enrique Navas-Suárez. Características e possíveis fatores de risco em cervos neotropicais com histórico de trauma e encaminhados ao Laboratório de Patologia Comparada de Animais Selvagens–LAPCOM, FMVZ, USP, Brasil. Revista de Educação Continuada em Medicina Veterinária e Zootecnia do CRMV-SP. 2016; 14:51–2. Available from: https://www.revistamvez-crmvsp.com.br/index.php/recmvz/article/view/31101.

[74] Lempp C, Jungwirth N, Grilo ML, Reckendorf A, Ulrich A, van Neer A, et al. Pathological findings in the red fox (Vulpes vulpes), stone marten (Martes foina) and raccoon dog (Nyctereutes procyonoides), with special emphasis on infectious and zoonotic agents in Northern Germany. PLoS One. 2017; 12:e0175469. Epub 2017/04/11. doi: 10.1371/journal.pone.0175469 . 28399176 PMC5388480

[75] Navas-Suárez PE, Díaz-Delgado J, Fernandes-Santos RC, Testa-José C, Silva R, Sansone M, et al. Pathological Findings in Lowland Tapirs (Tapirus terrestris) Killed by Motor Vehicle Collision in the Brazilian Cerrado. J Comp Pathol. 2019; 170:34–45. Epub 2019/06/12. doi: 10.1016/j.jcpa.2019.05.004 . 31375157

[76] Navas-Suárez P, Díaz-Delgado J, Matushima ER, Fávero CM, am Sánchez Sarmiento, Sacristán C, et al. A retrospective pathology study of two Neotropical deer species (1995–2015), Brazil: Marsh deer (Blastocerus dichotomus) and brown brocket deer (Mazama gouazoubira). PLoS One. 2018; 13:e0198670. Epub 2018/06/07. doi: 10.1371/journal.pone.0198670 . 29879222 PMC5991706

[77] Navas-Suárez PE, Sacristán C, Díaz-Delgado J, Yogui DR, Alves MH, Fuentes-Castillo D, et al. Pulmonary adiaspiromycosis in armadillos killed by motor vehicle collisions in Brazil. Sci Rep. 2021; 11:272. Epub 2021/01/11. doi: 10.1038/s41598-020-79521-6 . 33432031 PMC7801722

[78] Nettles VF, Quist CF, Lopez RR, Wilmers TJ, Frank P, Roberts W, et al. Morbidity and mortality factors in key deer (Odocoileus virginianus clavium). J Wildl Dis. 2002; 38:685–92. doi: 10.7589/0090-3558-38.4.685 . 12528433

[79] Rodriguez-Villar S, Fife A, Baldwin C, Warne RR. Antibiotic-resistant hypervirulent Klebsiella pneumoniae causing community- acquired liver abscess: an emerging disease. Oxf Med Case Reports. 2019; 2019:omz032. Epub 2019/05/31. doi: 10.1093/omcr/omz032 . 31198568 PMC6544431

[80] Greig J, Rajić A, Young I, Mascarenhas M, Waddell L, LeJeune J. A scoping review of the role of wildlife in the transmission of bacterial pathogens and antimicrobial resistance to the food Chain. Zoonoses Public Health. 2015; 62:269–84. Epub 2014/08/30. doi: 10.1111/zph.12147 . 25175882

[81] Heaton CJ, Gerbig GR, Sensius LD, Patel V, Smith TC. Staphylococcus aureus Epidemiology in Wildlife: A Systematic Review. Antibiotics (Basel). 2020; 9. Epub 2020/02/18. doi: 10.3390/antibiotics9020089 . 32085586 PMC7168057

[82] Mulani MS, Kamble EE, Kumkar SN, Tawre MS, Pardesi KR. Emerging Strategies to Combat ESKAPE Pathogens in the Era of Antimicrobial Resistance: A Review. Front Microbiol. 2019; 10:539. Epub 2019/04/01. doi: 10.3389/fmicb.2019.00539 . 30988669 PMC6452778

[83] Tacconelli E, Carrara E, Savoldi A, Harbarth S, Mendelson M, Monnet DL, et al. Discovery, research, and development of new antibiotics: the WHO priority list of antibiotic-resistant bacteria and tuberculosis. The Lancet Infectious Diseases. 2018; 18:318–27. doi: 10.1016/S1473-3099(17)30753-3 29276051

[84] Blanco-Peña K, Esperón F, Torres-Mejía AM, La Torre A de, La Cruz E de, Jiménez-Soto M. Antimicrobial Resistance Genes in Pigeons from Public Parks in Costa Rica. Zoonoses Public Health. 2017; 64:e23–e30. Epub 2017/02/24. doi: 10.1111/zph.12340 . 28233464 PMC5655739

[85] Grillet ME, Hernández-Villena JV, Llewellyn MS, Paniz-Mondolfi AE, Tami A, Vincenti-Gonzalez MF, et al. Venezuela’s humanitarian crisis, resurgence of vector-borne diseases, and implications for spillover in the region. The Lancet Infectious Diseases. 2019; 19:e149–e161. doi: 10.1016/S1473-3099(18)30757-6 30799251

[86] World Health Organization. A global brief on vector-borne diseases. Switzerland: WHO 2014. Available from: https://apps.who.int/iris/bitstream/handle/10665/111008/WHO_DCO_WHD_2014.1_eng.pdf.

[87] Dujardin J-C, Herrera S, do Rosario V, Arevalo J, Boelaert M, Carrasco HJ, et al. Research priorities for neglected infectious diseases in Latin America and the Caribbean region. PLoS Negl Trop Dis. 2010; 4:e780. Epub 2010/10/26. doi: 10.1371/journal.pntd.0000780 . 21049009 PMC2964298

[88] Montenegro VM, Bonilla MC, Kaminsky D, Romero-Zúñiga JJ, Siebert S, Krämer F. Serological detection of antibodies to *Anaplasma* spp., *Borrelia burgdorferi sensu lato* and *Ehrlichia canis* and of *Dirofilaria immitis* antigen in dogs from Costa Rica. Vet Parasitol. 2017; 236:97–107. Epub 2017/02/14. doi: 10.1016/j.vetpar.2017.02.009 . 28288773

[89] Rojas A, Rojas D, Montenegro VM, Baneth G. Detection of Dirofilaria immitis and other arthropod-borne filarioids by an HRM real-time qPCR, blood-concentrating techniques and a serological assay in dogs from Costa Rica. Parasit Vectors. 2015; 8:170. Epub 2015/03/23. doi: 10.1186/s13071-015-0783-8 . 25851920 PMC4377020

[90] León B, Käsbohrer A, Hutter SE, Baldi M, Firth CL, Romero-Zúñiga JJ, et al. National Seroprevalence and Risk Factors for Eastern Equine Encephalitis and Venezuelan Equine Encephalitis in Costa Rica. J Equine Vet Sci. 2020; 92:103140. Epub 2020/06/02. doi: 10.1016/j.jevs.2020.103140 . 32797803

[91] Calderón-Arguedas O, Troyo A, Solano ME, Avendaño A, Beier JC. Urban mosquito species (Diptera: Culicidae) of dengue endemic communities in the Greater Puntarenas area, Costa Rica. Rev Biol Trop. 2009; 57:1223–34. doi: 10.15517/rbt.v57i4.5459 . 20073347 PMC2832709

[92] Brenes R, Beaver PC, Monge E, Zamora L. Pulmonary dirofilariasis in a Costa Rican man. Am J Trop Med Hyg. 1985; 34:1142–3. doi: 10.4269/ajtmh.1985.34.1142 . 3834799

[93] Beaver PC, Brenes R, Ardon J. Dirofilaria from the index finger of a man in Costa Rica. Am J Trop Med Hyg. 1986; 35:988–90. doi: 10.4269/ajtmh.1986.35.988 3766857

[94] Olivo CA, Gundacker ND, Murillo J, Weiss SD, Suarez D, Suarez JA. Subcutaneous Dirofilariasis in a Returning Traveler From Costa Rica. Infect Dis Clin Pract. 2019; 27:58–60. doi: 10.1097/IPC.0000000000000679

[95] Johnson G, Nemeth N, Hale K, Lindsey N, Panella N, Komar N. Surveillance for West Nile virus in American white pelicans, Montana, USA, 2006–2007. Emerging Infect Dis. 2010; 16:406–11. doi: 10.3201/eid1603.090559 . 20202414 PMC3322008

[96] Johnson G, Panella N, Hale K, Komar N. Detection of West Nile virus in stable flies (Diptera: Muscidae) parasitizing juvenile American white pelicans. J Med Entomol. 2010; 47:1205–11. doi: 10.1603/me10002 . 21175073

[97] Piche M, Alfaro A, Jiménez-Soto M, Murcia P, Jiménez C. Caracterización molecular de dos brotes de Distemper Canino en animales de vida silvestre en Costa Rica. Ciencias Veterinarias. 2019; 36:38. doi: 10.15359/rcv.36-3.27

[98] Kapil S, Yeary TJ. Canine Distemper spillover in domestic dogs from urban wildlife. Vet Clin North Am Small Anim Pract. 2011; 41:1069–86. doi: 10.1016/j.cvsm.2011.08.005 . 22041204 PMC7132517

[99] Viana M, Cleaveland S, Matthiopoulos J, Halliday J, Packer C, Craft ME, et al. Dynamics of a morbillivirus at the domestic-wildlife interface: Canine Distemper virus in domestic dogs and lions. Proc Natl Acad Sci U S A. 2015; 112:1464–9. Epub 2015/01/20. doi: 10.1073/pnas.1411623112 . 25605919 PMC4321234

[100] Rendon-Marin S, Martinez-Gutierrez M, Suarez JA, Ruiz-Saenz J. Canine Distemper Virus (CDV) Transit Through the Americas: Need to Assess the Impact of CDV Infection on Species Conservation. Front Microbiol. 2020; 11:810. Epub 2020/05/21. doi: 10.3389/fmicb.2020.00810 . 32508760 PMC7253583

[101] Beineke A, Baumgärtner W, Wohlsein P. Cross-species transmission of Canine Distemper virus-an update. One Health. 2015; 1:49–59. Epub 2015/09/13. doi: 10.1016/j.onehlt.2015.09.002 . 28616465 PMC5462633

[102] Vargas M. Informe técnico Evaluación de test de Morera según resultados del Centro Nacional de Referencia de Parasitología- Inciensa. Costa Rica enero 2012—abril 2020. Costa Rica: Instituto Costarricense de Investigación y Enseñanza en Nutrición y Salud. 2020. Available from: https://www.inciensa.sa.cr/vigilancia_epidemiologica/informes_vigilancia/2020/CNR%20Parasitologia/Informe%20Tecnico%20A.%20costarricensis.pdf.

[103] Wise ME, Sorvillo FJ, Shafir SC, Ash LR, Berlin OG. Severe and fatal central nervous system disease in humans caused by Baylisascaris procyonis, the common roundworm of raccoons: a review of current literature. Microbes Infect. 2005; 7:317–23. Epub 2005/01/08. doi: 10.1016/j.micinf.2004.12.005 . 15715975

[104] Mathison BA, Bishop HS, Sanborn CR, Dos Santos Souza S, Bradbury R. *Macracanthorhynchus ingens* Infection in an 18-Month-Old Child in Florida: A Case Report and Review of Acanthocephaliasis in Humans. Clin Infect Dis. 2016; 63:1357–9. Epub 2016/08/07. doi: 10.1093/cid/ciw543 . 27501844

[105] Badilla X, Pérez-Herra V, Quirós L, Morice A, Jiménez E, Sáenz E, et al. Human rabies: a reemerging disease in Costa Rica. Emerging Infect Dis. 2003; 9:721–3. doi: 10.3201/eid0906.020632 . 12781014 PMC3000141

[106] Hernandez SM, Peters VE, Weygandt PL, Jimenez C, Villegas P, O’Connor B, et al. Do shade-grown coffee plantations pose a disease risk for wild birds. Ecohealth. 2013; 10:145–58. Epub 2013/05/02. doi: 10.1007/s10393-013-0837-3 . 23636482

[107] Hernández-Mora G, Bonilla-Montoya R, Barrantes-Granados O, Esquivel-Suárez A, Montero-Caballero D, González-Barrientos R, et al. Brucellosis in mammals of Costa Rica: An epidemiological survey. PLoS One. 2017; 12:e0182644. Epub 2017/08/09. doi: 10.1371/journal.pone.0182644 . 28793352 PMC5549988

[108] Lipkin WI. The changing face of pathogen discovery and surveillance. Nat Rev Microbiol. 2013; 11:133–41. Epub 2013/01/03. doi: 10.1038/nrmicro2949 . 23268232 PMC4098826

[109] Gardy JL, Loman NJ. Towards a genomics-informed, real-time, global pathogen surveillance system. Nat Rev Genet. 2018; 19:9–20. Epub 2017/11/13. doi: 10.1038/nrg.2017.88 . 29129921 PMC7097748

[110] Mishra N, Fagbo SF, Alagaili AN, Nitido A, Williams SH, Ng J, et al. A viral metagenomic survey identifies known and novel mammalian viruses in bats from Saudi Arabia. PLoS One. 2019; 14:e0214227. Epub 2019/04/10. doi: 10.1371/journal.pone.0214227 . 30969980 PMC6457491

[111] Gu W, Deng X, Lee M, Sucu YD, Arevalo S, Stryke D, et al. Rapid pathogen detection by metagenomic next-generation sequencing of infected body fluids. Nat Med. 2021; 27:115–24. Epub 2020/11/09. doi: 10.1038/s41591-020-1105-z . 33169017 PMC9020267

[112] Lawson B, Neimanis A, Lavazza A, López-Olvera JR, Tavernier P, Billinis C, et al. How to Start Up a National Wildlife Health Surveillance Programme. Animals (Basel). 2021; 11. Epub 2021/08/30. doi: 10.3390/ani11092543 . 34573509 PMC8467383

