# Supplementary material for: Passive epidemiological surveillance in wildlife in Costa Rica identifies pathogens of zoonotic and conservation importance
Source: PLoS One. 2022 Sep 26;17(9):e0262063. doi: 10.1371/journal.pone.0262063 (PMC9512195; doi:10.1371/journal.pone.0262063)
Supplement: S1 Table — (DOCX) [file pone.0262063.s001.docx]

**Supporting information**

**S.2 Table: Analyzed animals: Case number, common name, age, gender, body condition, macroscopical and histological lesions, cause of death and diagnosed infectious agents.**

| **Case number** | **common name** | **scientific name** | **Age** | **Gender** | **Body Condition** | **Main macroscopical and histological lesions** | **Cause of death** | **Diagnosed infectious agents (technique)** |
| --- | --- | --- | --- | --- | --- | --- | --- | --- |
| 1 | Raccoon | *Procyon lotor* | Adult | Male | Regular | Suppurative bronchointerstitial pneumonia. Diffuse catarrhal duodeno jejunitis. Bilateral suppurative conjunctivitis. Non-suppurative meningoencephalitis, extensive demyelination, and intracytoplasmic inclusion bodies. | Infectious disease | CDV (RT-PCR) |
| 2 | Raccoon | *Procyon lotor* | Juvenile | Male | Good | Suppurative bronchointerstitial pneumonia. Lymphoplasmacytic cholangiohepatitis. Bilateral suppurative conjunctivitis. Non-suppurative meningoencephalitis, extensive demyelination, and intracytoplasmic inclusion bodies. | Infectious disease | CDV (RT-PCR) |
| 3 | Squirrel monkey | *Saimiri oerstedii* | Adult | Male | Good | Laceration of internal organs. Multifocal transmural pyogranulomatous and hemorrhagic colitis. | Traumatic  (inter-species attack) | *Prosthenorchis* spp. (Morphology) |
| 4 | Raccoon | *Procyon lotor* | Juvenile | Female | Poor | Suppurative bronchointerstitial pneumonia. Diffuse catarrhal duodeno jejunitis. Suppurative interstitial nephritis. Non-suppurative meningoencephalitis, extensive demyelination, and intracytoplasmic inclusion bodies. Splenic lymphoid depletion. | Infectious disease | CDV (RT-PCR) |
| 5 | Two-toed sloth | *Choloepus hoffmanni* | Adult | Female | Good | Electrothermal burn. Bronchointerstitial lymphoplasmacytic pneumonia with multifocal hemorrhages. Focal suppurative and hemorrhagic cystitis. | Traumatic  (Electric shock) | __ |
| 6 | Spider monkey | *Ateles geoffroyi* | Adult | Female | Good | Electrothermal burn. Generalized jaundice. Fibrinosuppurative and hemorrhagic pleuropneumonia. Non-suppurative meningoencephalitis. | Traumatic  (Electric shock) | *Dipetalonema* spp. (Morphology) |
| 7 | Howler monkey | *Alouatta palliata* | Adult | Female | Regular | Hip fracture. Anasarca. Focal eosinophilic cholangiohepatitis. | Traumatic  (Traffic accident) | *Controrchis* spp. (Morphology) |
| 8 | Grey Fox | *Urocyon cinereoargenteus* | Adult | Male | Good | Lymphoplasmacytic interstitial pneumonia with intracytoplasmic inclusions and secondary bacteria and foci of granulomatous inflammation. Lymphoplasmacytic cholangiohepatitis. Florida keratopathy. Focal Lymphoplasmacytic cystitis. Non-suppurative meningoencephalitis, extensive demyelination, and intracytoplasmic inclusion bodies. | Infectious disease | CDV (RT-PCR) |
| 9 | Raccoon | *Procyon lotor* | Juvenile | Female | Regular | Cranioencephalic Trauma. Multifocal transmural pyogranulomatous and hemorrhagic colitis. | Traumatic  (Traffic accident) | *Prosthenorchis* spp. (Morphology)  *Macracanthorhynchus* spp. (Morphology) |
| 10 | Ant-eater | *Tamandua mexicana* | Adult | Female | Regular | Central nervous system hemorrhage. | Traumatic  (Traffic accident) | *Gyganthorhynchus* spp. (Morphology) |
| 15 | Whitetail deer | *Odocoileus virginianus* | Adult | Female | Good | Necrotizing suppurative myositis. Multifocal hemorrhage pulmonary. Focal extensive Hemorrhagic pericarditis. Multifocal extensive hepatic necrosis. | Infectious disease | *Clostridium perfringens.* (VITEK)  *Sarcocystis* spp. (Morphology)  *Setaria* spp. (Morphology) |
| 16 | Muscovy duck | *Cairina moschata* | Adult | Female | Regular | Petechial hemorrhages in epicardium. Periportal multifocal hepatic necrosis. | suspected poisoning | __ |
| 17 | Muscovy duck | *Cairina moschata* | Adult | Female | Regular | Petechial hemorrhages in epicardium. Periportal multifocal hepatic necrosis. | suspected poisoning | __ |
| 18 | Squirrel monkey | *Saimiri oerstedii* | Adult | Male | Regular | Cranioencephalic Trauma. Multifocal transmural pyogranulomatous and hemorrhagic colitis. Suppurative peritonitis. | Traumatic  (Traffic accident) | *Prosthenorchis* spp. (Morphology) |
| 19 | Three-toed sloth | *Bradypus variegatus* | Adult | Male | Regular | Bilateral suppurative rhinitis. Diffuse fibrinosuppurative and hemorrhagic pleurobronchopneumonia. Focal suppurative and hemorrhagic cystitis. Bilateral suppurative conjunctivitis. | Undetermined | __ |
| 20 | Nine-banded armadillo | *Dasypus novemcinctus* | Adult | Female | Good | Multifocal perforating wounds. Multifocal suppurative necrotizing myositis. | Traumatic  (inter-species attack) | __ |
| 21 | Grey Fox | *Urocyon cinereoargenteus* | Adult | Male | Regular | Lymphoplasmacytic interstitial pneumonia with intracytoplasmic inclusions and secondary bacteria and foci of granulomatous inflammation. Lymphoplasmacytic cholangiohepatitis. Non-suppurative meningoencephalitis, extensive demyelination, and intracytoplasmic inclusion bodies. | Infectious disease | CDV (RT-PCR) |
| 22 | Howler monkey | *Alouatta palliata* | Adult | Female | Regular | Central nervous system hemorrhage. Pyogranulomatous and hemorrhagic pneumonia. Mesenteric congestion with intravascular microfilariae. Multifocal lymphohistiocytic necrotizing hepatitis. Lymphohistiocytic colitis.  Bilateral multifocal Renal mineralization. Multifocal lymphohistiocytic Necrotizing splenitis. | Traumatic  (Traffic accident) | *Dipetalonema* spp. (Morphology)  *Trypanoxyuris* spp. (Morphology) |
| 23 | Capuchin monkey | *Cebus imitator* | Adult | Male | Regular | Laceration of internal organs. Diffuse eosinophilic pyogranulomatous and hemorrhagic bronchopneumonia with presence of nematodes. Multifocal Eosinophilic lymphoplasmacytic necrotizing hepatitis. Unilateral testicular hypoplasia. Multifocal splenic lymphoid follicular hyperplasia. | Traumatic  (Traffic accident) | *Dipetalonema* spp. (Morphology)  *Filariopsis* spp. (Morphology) |
| 25 | Whitetail deer | *Odocoileus virginianus* | Juvenile | Female | Good | Cranioencephalic Trauma. | Traumatic  (Traffic accident) | __ |
| 27 | Porcupine | *Sphiggurus mexicanus* | Adult | Female | Regular | Multifocal perforating wounds. Focal suppurative necrotizing myositis. | Traumatic  (inter-species attack) | __ |
| 28 | White-nosed Coati | *Nasua narica* | Adult | Female | Good | Laceration of internal organs. Diffuse fibrinosuppurative bronchointerstitial pneumonia. Pulmonary congestion with intravascular filariae. Multifocal transmural pyogranulomatous and hemorrhagic colitis. Suppurative peritonitis. Bilateral diffuse suppurative and hemorrhagic interstitial nephritis. Bilateral suppurative conjunctivitis. | Traumatic  (Traffic accident) | *Dirofilaria* spp. (Morphology)  *Prosthenorchis* spp. (Morphology) |
| 29 | White-nosed Coati | *Nasua narica* | Adult | Female | Good | Cranioencephalic Trauma. Diffuse fibrinosuppurative Bronchointerstitial pneumonia. Pulmonary congestion with intravascular filariae. Multifocal pyogranulomatous and eosinophilic mesenteric vasculitis. Multifocal transmural pyogranulomatous and hemorrhagic colitis. Suppurative peritonitis. | Traumatic  (Traffic accident) | *Angiostrongylus* spp. (Morphology)  *Dirofilaria* spp. (Morphology)  *Prosthenorchis* spp. (Morphology) |
| 30 | Howler monkey | *Alouatta palliata* | Juvenile | Male | Poor | Laceration of internal organs. Palatoschisis. Hepatic lipidosis. | Traumatic  (intra-species attack) | __ |
| 31 | Western osprey | *Pandion haliaetus* | Juvenile | Male | Good | Myxomatous valve degeneration. | degenerative illness | __ |
| 32 | Howler monkey | *Alouatta palliata* | Adult | Male | Good | Multiple exposed fractures. | Traumatic  (Traffic accident) | __ |
| 34 | Opossum | *Didelphis marsupialis* | Adult | Male | Regular | Cranioencephalic Trauma. Diffuse eosinophilic fibrinosuppurative abscessing pleurobronchopneumonia. Multifocal histiocytic and lymphoplasmacytic myocarditis. Focal mural pyogranulomatous gastritis. | Traumatic  (Traffic accident) | *Trypanosoma cruzi*. (PCR)  *Gnathostoma* spp. (Morphology)  *Cruzia* spp. (Morphology) |
| 35 | Raccoon | *Procyon lotor* | Adult | Male | Good | Cranioencephalic Trauma. Centrilobular focal lymphocytic hepatitis. Unilateral testicular hypoplasia. Multifocal splenic lymphoid follicular hyperplasia. | Traumatic  (Traffic accident) | *Baylisascaris* spp. (Morphology) |
| 36 | White-nosed Coati | *Nasua narica* | Adult | Male | Regular | Laceration of internal organs. Diffuse fibrinosuppurative Bronchointerstitial pneumonia. Pulmonary congestion with intravascular filariae. Multifocal pyogranulomatous and eosinophilic mesenteric vasculitis. Multifocal transmural pyogranulomatous and hemorrhagic colitis. Bilateral diffuse suppurative and hemorrhagic interstitial nephritis. | Traumatic  (Traffic accident) | *Angiostrongylus* spp. (Morphology)  *Dirofilaria* spp. (Morphology)  *Prosthenorchis* spp. (Morphology) |
| 39 | Howler monkey | *Alouatta palliata* | Adult | Female | Good | Generalized pyogranulomatous and hemorrhagic interstitial pneumonia with intralesional bradyzoite pseudocysts and anthracosis. Multifocal pyogranulomatous necrotizing ulcerative and hemorrhagic jejunitis with intramural bacterial clumps. Bilateral diffuse histiocytic and lymphoplasmacytic interstitial nephritis with mucinous tubular degeneration. Diffuse pyogranulomatous hepatitis with intralesional bradyzoite pseudocysts. Multifocal perivascular histiocytic and lymphoplasmacytic encephalitis with leukoencephalomalacia. Diffuse granulomatous necrotizing splenitis. | Infectious disease | *Toxoplasma gondii.* (PCR)  *Controrchis* spp. (Morphology) |
| 40 | Spider monkey | *Ateles geoffroyi* | Adult | Male | Regular | Laceration of internal organs. Focal suppurative necrotizing myositis. Generalized fibrinosuppurative abscessing pleurobronchopneumonia. Multifocal suppurative endocarditis. Fibrinosuppurative peritonitis abscessing. Diffuse suppurative jejunitis. Multifocal pyogranulomatous hepatitis. Bilateral diffuse suppurative nephritis. Focal pyogranulomatous lymphadenitis. | Traumatic  (intra-species attack) | *Staphylococcus aureus.* (VITEK) |
| 41 | Jaguarundi | *Herpailurus yagouaroundi* | Adult | Male | Good | Laceration of internal organs. | Traumatic  (Traffic accident) | *Amblyomma ovale* (Morphology) |
| 42 | Ocelot | *Leopardus pardalis* | Adult | Male | Good | Cranioencephalic Trauma. | Traumatic  (Traffic accident) | *Spirometra* spp. (Morphology)  *Oncicola* spp. (Morphology)  *Taenia* spp. (Morphology) |
| 43 | Howler monkey | *Alouatta palliata* | Juvenile | Male | Poor | Generalized pyogranulomatous and hemorrhagic interstitial pneumonia with intralesional bradyzoite pseudocysts and anthracosis. Multifocal pyogranulomatous necrotizing ulcerative and hemorrhagic jejunitis with intramural bacterial clumps. Bilateral diffuse histiocytic and lymphoplasmacytic interstitial nephritis with mucinous tubular degeneration. Diffuse pyogranulomatous hepatitis with intralesional bradyzoite pseudocysts. Multifocal perivascular histiocytic and lymphoplasmacytic encephalitis with leukoencephalomalacia. Diffuse granulomatous necrotizing splenitis. | Infectious disease | *Toxoplasma gondii.* (PCR) |
| 44 | Two-toed sloth | *Choloepus hoffmanni* | Juvenile | Female | Good | Diffuse fibrinosuppurative and hemorrhagic bronchopneumonia. Bilateral Suppurative rhinitis. Bilateral Suppurative conjunctivitis. | Undetermined | __ |
| 45 | Raccoon | *Procyon lotor* | Adult | Male | Poor | Myxomatous valve degeneration. Ascites and Midzonal extensive hepatic necrosis. Fibrosarcoma. | degenerative illness | __ |
| 46 | Squirrel monkey | *Saimiri oerstedii* | Adult | Female | Poor | Multifocal transmural pyogranulomatous and hemorrhagic ileocolitis. Suppurative peritonitis. Focal extensive suppurative abscessing hepatitis. Unilateral focal extensive suppurative abscessing nephritis. | Infectious disease | *Escherichia coli.* (VITEK)  *Prosthenorchis* spp. (Morphology) |
| 47 | Two-toed sloth | *Choloepus hoffmanni* | Adult | Male | Good | Electrothermal burn. Focal suppurative abscessing osteomyelitis. Diffuse fibrinosuppurative pleurobronchopneumonia. | Traumatic  (Electric shock) | *Staphylococcus aureus.* (VITEK) |
| 48 | Jaguarundi | *Herpailurus yagouaroundi* | Adult | Male | Good | Laceration of internal organs. Multifocal eosinophilic granulomatous interstitial pneumonia with intralesional nematodes. Multifocal granulomatous gastritis with intralesional nematodes. Diffuse catarrhal duodeno jujunitis. | Traumatic  (Traffic accident) | *Cylicospirura* spp. (Morphology)  *Spirometra* spp. (Morphology)  *Oslerus* spp. (Morphology)  *Taenia* spp. (Morphology)  *Spirometra* spp. (Morphology) |
| 49 | Howler monkey | *Alouatta palliata* | Juvenile | Male | Poor | Multifocal lymphoplasmacytic and eosinophilic necrotizing typhlocolitis. Periportal and centrilobular multifocal histiocytic and lymphoplasmacytic hepatitis. | Undetermined | __ |
| 50 | Howler monkey | *Alouatta palliata* | Juvenile | Male | Poor | Multifocal pyogranulomatous erosive rhinopharyngitis. Diffuse lymphoplasmacytic interstitial pneumonia. Multifocal lymphoplasmacytic and eosinophilic necrotizing typhlocolitis. Periportal and centrilobular multifocal lymphoplasmacytic hepatitis. Multifocal splenic lymphoid follicular hyperplasia. | Undetermined | __ |
| 51 | Margay | *Leopardus weidii* | Adult | Male | Good | Multifocal eosinophilic granulomatous pneumonia with intralesional nematodes. Midzonal diffuse hepatic necrosis. | Suspected poisoning | *Oslerus* spp. (Morphology) |
| 52 | Squirrel monkey | *Saimiri oerstedii* | Adult | Male | Poor | Multifocal suppurative ulcerative dermatitis. Multifocal transmural pyogranulomatous and hemorrhagic colitis. Suppurative peritonitis. | infectious disease | *Prosthenorchis* spp. (Morphology) |
| 53 | Squirrel monkey | *Saimiri oerstedii* | Juvenile | Male | Poor | Hip fracture. Diffuse lymphoplasmacytic interstitial pneumonia. Multifocal transmural pyogranulomatous and hemorrhagic colitis. | Traumatic  (Traffic accident) | *Prosthenorchis* spp. (Morphology) |
| 54 | Black hawk-eagle | *Spizaetus tyrannus* | Juvenile | Male | Good | Multiple exposed fractures. Multifocal eosinophilic proventriculitis. | Traumatic  (Traffic accident) | *Procyrnea* spp. (Morphology) |
| 57 | Woolly opossums | *Caluromys derpianus* | Adult | Female | Good | Mild lymphoplasmacytic bronchoalveolar pneumonia. | Undetermined | __ |
| 58 | Opossum | *Didelphis marsupialis* | Adult | Female | Good | Cranioencephalic Trauma. Multifocal histiocytic and lymphoplasmacytic myocarditis. | Traumatic  (Traffic accident) | *Trypanosoma cruzi.* (PCR) |
| 59 | Howler monkey | *Alouatta palliata* | Adult | Male | Regular | Focal suppurative abscessing osteomyelitis. Unilateral diffuse suppurative rhinitis. Focal extensive fibrinosuppurative abscessing pleurobronchopneumonia. Diffuse suppurative necrotizing typhlocolitis. Multifocal pyogranulomatous cholangiohepatitis. Unilateral suppurative conjunctivitis. Unilateral suppurative uveitis. | Infectious disease | *Klebsiella pneumoniae.* (VITEK)  *Conthorchis* spp. (Morphology) |
| 60 | Capuchin monkey | *Cebus imitator* | Adult | Male | Regular | Multifocal granulomatous and eosinophilic interstitial pneumonia. Multifocal eosinophilic ulcerative typhlocolitis. | Infectious disease | *Filariopsis* spp. (Morphology) |
| 61 | Brown-throated sloth | *Bradypus variegatus* | Adult | Male | Poor | Multifocal perforating wounds. Focal suppurative abscessing necrotizing myositis. | Traumatic  (intra-species attack) | __ |
| 62 | Whitetail deer | *Odocoileus virginianus* | Adult | Female | Good | Hip fracture. | Traumatic  (Traffic accident) | *Ixodes affinis.* (Morphology) |
| 63 | Pelican | *Pelicanus occidentalis* | Adult | Male | Poor | Diffuse lymphoplasmacytic interstitial pneumonia. Diffuse pyogranulomatous and eosinophilic ulcerative proventriculitis. Diffuse pyogranulomatous and eosinophilic ulcerative ventriculitis. Multifocal lymphoplasmacytic necrotizing hepatitis. Congestive hepatomegaly.  Bilateral lymphoplasmacytic Interstitial nephritis with intralesional trematodes. | Infectious disease | Flavivirus (RT-PCR)  *Contracaecum* spp. (Morphology) |
| 64 | Opossum | *Didelphis marsupialis* | Juvenile | Female | Poor | Multifocal eosinophilic dermatitis. Diffuse lymphohistiocytic bronchointerstitial pneumonia. Diffuse catarrhal duodeno jejunitis. Multifocal eosinophilic meningoencephalitis. | Infectious disease | *Angiostrongylus* spp. (Morphology)  *Cruzia* spp. (Morphology)  *Ixodes affinis.* (Morphology)  *Amblyomma* spp. (Morphology)  *Ornithodoros* spp. (Morphology) |
| 65 | Squirrel monkey | *Saimiri oerstedii* | Adult | Female | Poor | Multifocal transmural pyogranulomatous and hemorrhagic colitis. | Infectious disease | *Prosthenorchis* spp. (Morphology) |
| 66 | Lesson's motmot | *Momotus lessonii* | Adult | Male | Regular | Focal extensive granulomatous bronchopneumonia. | Undetermined | __ |
| 67 | Squirrel monkey | *Saimiri oerstedii* | Adult | Female | Good | Electrothermal burn. Multifocal transmural pyogranulomatous and hemorrhagic colitis. | Traumatic  (Electric shock) | *Prosthenorchis* spp. (Morphology) |
| 68 | Aracari | *Pteroglossus Frantzii* | Adult | Male | Regular | Multiple exposed fractures. | Traumatic  (Traffic accident) | *Brueelia* spp. (Morphology) |
| 69 | Raccoon | *Procyon lotor* | Adult | Male | Regular | Suppurative bronchointerstitial pneumonia. Diffuse catarrhal duodeno jejunitis. Bilateral suppurative conjunctivitis. non-suppurative meningoencephalitis, extensive demyelination, and intracytoplasmic inclusion bodies. | Infectious disease | CDV (RT-PCR) |
| 70 | Raccoon | *Procyon lotor* | Adult | Male | Regular | Suppurative bronchointerstitial pneumonia. Bilateral suppurative conjunctivitis. non-suppurative meningoencephalitis, extensive demyelination, and intracytoplasmic inclusion bodies. | Infectious disease | CDV (RT-PCR) |
| 71 | Howler monkey | *Alouatta palliata* | Adult | Female | Poor | Multifocal perforating wounds. Multifocal lymphoplasmacytic and histiocytic bronchoalveolar pneumonia. Diffuse catarrhal colitis. Periportal multifocal lymphoplasmacytic hepatitis. | Traumatic  (inter-species attack) | *Trypanoxyuris* spp. (Morphology) |
| 72 | Pelican | *Pelicanus occidentalis* | Adult | Male | Poor | Diffuse pseudomembranous lymphoplasmacytic ulcerative tracheitis. Diffuse lymphoplasmacytic interstitial pneumonia. Diffuse pyogranulomatous and eosinophilic ulcerative proventriculitis. Diffuse pyogranulomatous and eosinophilic ulcerative ventriculitis. Multifocal lymphoplasmacytic necrotizing hepatitis. Congestive hepatomegaly. Bilateral lymphoplasmacytic Interstitial nephritis with intralesional trematodes. | Infectious disease | Flavivirus (RT-PCR)  *Contracaecum* spp. (Morphology)  *Cyathostoma* spp. (Morphology)  *Renicola* spp. (Morphology)  *Pectinopygus* spp. (Morphology) |
| 73 | Squirrel monkey | *Saimiri oerstedii* | Adult | Male | Regular | Cranioencephalic Trauma. Multifocal transmural pyogranulomatous and hemorrhagic colitis. Suppurative peritonitis. | Traumatic  (Traffic accident) | *Prosthenorchis* spp. (Morphology) |
| 74 | Ant-eater | *Tamandua mexicana* | Juvenile | Male | Good | Electrothermal burn. Multifocal granulomatous and eosinophilic interstitial pneumonia with intralesional microfilariae. | Traumatic  (Electric shock) | *Dipetalonema* spp. (Morphology)  *Amblyomma nodosum.* (Morphology) |
| 75 | Ant-eater | *Tamandua mexicana* | Adult | Female | Good | Central nervous system hemorrhage. | Traumatic  (Traffic accident) | *Amblyomma nodosum.* (Morphology) |
| 76 | Nine-banded armadillo | *Dasypus novemcinctus* | Juvenile | Female | Good | Multifocal perforating wounds. Multifocal granulomatous interstitial pneumonia. | Traumatic  (inter-species attack) | __ |
| 77 | Two-toed sloth | *Choloepus hoffmanni* | Adult | Female | Good | Electrothermal burn. | Traumatic  (Electric shock) | *Amblyomma nodosum.* (Morphology) |
| 78 | Whitetail deer | *Odocoileus virginianus* | Juvenile | Male | Good | Cranioencephalic Trauma. | Traumatic  (Traffic accident) | __ |
| 79 | Porcupine | *Sphiggurus mexicanus* | Adult | Female | Poor | Severe chronic generalized eosinophilic dermatitis. Parakeratosis. Multifocal suppurative necrotizing myositis. Focal extensive suppurative abscessing pleurobronchopneumonia. Bilateral suppurative conjunctivitis. Bilateral suppurative otitis. | Infectious disease | *Sarcoptex* spp. (Morphology)  *Trueperella pyogenes* |
| 80 | Tayra | *Eira barbara* | Adult | Male | Good | Diffuse pyogranulomatous and eosinophilic necrotizing pneumonia with intralesional nematodes. Diffuse lymphoplasmacytic meningitis. | Undetermined | *Angiostrongylus* spp. (Morphology) |
| 81 | Opossum | *Didelphis marsupialis* | Adult | Female | Poor | Multifocal perforating wounds. Diffuse lymphohistiocytic interstitial pneumonia with aggregates of bacterial. Multifocal lymphoplasmacytic myocarditis. Periportal multifocal lymphoplasmacytic hepatitis. Bilateral focal suppurative interstitial nephritis. | Traumatic  (inter-species attack) | *Trypanosoma cruzi.* (PCR)  *Cruzia* spp. (Morphology) |
| 82 | Agouti | *Dasyprocta Punctata* | Juvenile | Female | Good | Multifocal pyogranulomatous interstitial pneumonia. | Undetermined | __ |
| 83 | Jabiru | *Jabiru mycteria* | Juvenile | Male | Good | Multifocal lymphocytic interstitial pneumonia. Multifocal granulomatous myocarditis with crystal deposits. Bilateral multifocal granulomatous nephritis with crystal deposits. | Undetermined | __ |
| 84 | Capuchin monkey | *Cebus imitator* | Adult | Male | Regular | Diffuse pyogranulomatous and eosinophilic cystic bronchointerstitial pneumonia with intralesional nematodes. Multifocal transmural pyogranulomatous and hemorrhagic colitis. | Infectious disease | *Dipetalonema* spp. (Morphology)  *Prosthenorchis* spp. (Morphology)  *Filariopsis* spp. (Morphology) |
| 85 | White-nosed Coati | *Nasua narica* | Adult | Male | Good | Hip fracture. Focal eosinophilic myositis. Multifocal pyogranulomatous and eosinophilic mesenteric vasculitis. Focal lymphoplasmacytic hepatitis. | Traumatic  (Traffic accident) | *Angiostrongylus* spp. (Morphology)  *Sarcocystis* spp. (Morphology) |
| 86 | White-nosed Coati | *Nasua narica* | Adult | Female | Good | Cranioencephalic Trauma. Diffuse lymphoplasmacytic Interstitial pneumonia. Pulmonary congestion with intravascular filariae. Multifocal pyogranulomatous and eosinophilic mesenteric vasculitis. Multifocal transmural pyogranulomatous and hemorrhagic colitis. | Traumatic  (Traffic accident) | *Angiostrongylus* spp. (Morphology)  *Dirofilaria* spp. (Morphology)  *Prosthenrochis* spp. (Morphology) |
| 87 | Capuchin monkey | *Cebus imitator* | Adult | Female | Poor | Laceration of internal organs. Multifocal pyogranulomatous and eosinophilic interstitial pneumonia with intralesional nematodes. Multifocal transmural pyogranulomatous and hemorrhagic colitis. Multifocal pyogranulomatous and eosinophilic hepatitis. | Traumatic  (Traffic accident) | *Dipetalonema* spp. (Morphology)  *Prostheorchis* spp. (Morphology)  *Filariopsis* spp. (Morphology) |
| 88 | Ant-eater | *Tamandua mexicana* | Adult | Female | Good | Laceration of internal organs. Focal extensive suppurative and hemorrhagic necrotizing myositis. Diffuse pyogranulomatous bronchopneumonia with foci of metastatic mineralization. Pulmonary congestion with intravascular filariae. Diffuse liver necrosis. Focal lymphoplasmacytic splenitis. | Traumatic  (inter-species attack) | *Dipetalonema* spp. (Morphology) |
| 89 | Coyote | *Canis latrans* | Adult | Male | Poor | Multiple exposed fractures. Generalized eosinophilic dermatitis. Focal suppurative abscessing myositis. Focal extensive fibrinosuppurative abscessing pleurobronchopneumonia with dystrophic calcification. Multifocal granulomatous aortic arteritis with dystrophic mineralization. Diffuse eosinophilic necrotizing duodeno jejunitis. Bilateral multifocal lymphoplasmacytic nephritis with dystrophic calcification. Bilateral suppurative otitis. | Traumatic  (Traffic accident) | *Staphylococcus aureus.* (VITEK)  *Ancylostoma* spp. (Morphology)  *Spirocerca lupi.* (Morphology) |
| 90 | White-nosed Coati | *Nasua narica* | Juvenile | Male | Poor | Suppurative bronchointerstitial pneumonia. Lymphoplasmacytic cholangiohepatitis. Bilateral suppurative conjunctivitis. Non-suppurative meningoencephalitis, extensive demyelination, and intracytoplasmic inclusion bodies. Splenic lymphoid depletion. | Infectious disease | CDV (RT-PCR) |
| 91 | White-nosed Coati | *Nasua narica* | Juvenile | Male | regular | Suppurative bronchointerstitial pneumonia. Non-suppurative meningoencephalitis, extensive demyelination, and intracytoplasmic inclusion bodies. Splenic lymphoid depletion. | Infectious disease | CDV (RT-PCR) |
| 92 | Grey Fox | *Urocyon cinereoargenteus* | Juvenile | Male | Poor | Suppurative bronchointerstitial pneumonia. Non-suppurative meningoencephalitis, extensive demyelination, and intracytoplasmic inclusion bodies. | Infectious disease | CDV (RT-PCR) |
| 93 | Kinkajou | *Potos Flavus* | Juvenile | Female | Good | Multifocal lymphoplasmacytic and hemorrhagic interstitial pneumonia. Midzonal multifocal lymphoplasmacytic hepatitis. Microphthalmia. Multifocal lymphoplasmacytic encephalitis. Splenic lymphoid depletion. | Undetermined | __ |
| 94 | Howler monkey | *Alouatta palliata* | Adult | Female | Poor | Electrothermal burn. Diffuse lymphoplasmacytic and histiocytic interstitial pneumonia. Eosinophilic erosive typhlocolitis. Focal eosinophilic cholangiohepatitis. | Traumatic  (Electric shock) | *Controrchis* spp. (Morphology)  *Trypanoxyuris* spp. (Morphology) |
| 95 | Ant-eater | *Tamandua mexicana* | Adult | Female | regular | Cranioencephalic Trauma. Midzonal and periportal diffuse liver necrosis. | Traumatic  (Traffic accident) | __ |
| 96 | Porcupine | *Sphiggurus mexicanus* | Juvenile | Male | Good | Multifocal lymphoplasmacytic and hemorrhagic interstitial pneumonia. Diffuse lymphocytic and hemorrhagic encephalitis. | Undetermined | __ |
