# Supplementary material for: Passive epidemiological surveillance in wildlife in Costa Rica identifies pathogens of zoonotic and conservation importance
Source: PLoS One. 2022 Sep 26;17(9):e0262063. doi: 10.1371/journal.pone.0262063 (PMC9512195; doi:10.1371/journal.pone.0262063)
Supplement: Attachment — Submitted filename: Response to reviewers.pdf [file pone.0262063.s004.pdf]

UNA
UNIVERSIDAD NACIONAL
COSTA RICA

Heredia, August 11th, 2022

Daniel Becker
Academic Editor
PLOS ONE

I am attaching the revised version of our manuscript: "Passive epidemiological surveillance in wildlife in Costa Rica identifies pathogens of zoonotic and conservation importance" by Fernando Aguilar-Vargas and Tamara Solorzano-Scott1 *et al.*, which we would like to resubmit for publication as a Research Article to PLoS ONE.

We want to acknowledge all the reviewers' comments since they have helped us shape our work and improve the manuscript. We have paid attention to all the concerns raised by both reviewers, made the changes when required, and highlighted all changes in yellow color in the text.

We appreciate the editor and referees' time and efforts in reviewing this manuscript and believe that the revised version will meet the journal publication requirements.

Sincerely yours,

ALEJANDRO JOSE ALFARO ALARCON (FIRMA)

Firmado digitalmente por ALEJANDRO JOSE ALFARO ALARCON (FIRMA)
Fecha: 2022.08.11 10:13:52 -06'00'

Dr. Alejandro Alfaro-Alarcón PhD.
Department of Anatomic Pathology
National University of Costa Rica
Head of Department

Response to reviewers

Reviewer 1

In their first submission, the authors only presented their results without proposing a feasible and standardized method to implement this tool. In this version, they provide more detail (exemplified in figure 1), but I am afraid that their suggestions are very country specific. If this manuscript is accepted, I suggest that it would be under the study type "Methods, software, databases, and tools", which should meet the criteria of utility, validation and availability (see https://journals.plos.org/plosone/s/submission-guidelines#loc-methods-software-databases-and-tools), and should be developed more broadly to propose a scheme for other countries with similar challenges. Although, their epidemiological findings are

interesting by themself, they are not the real focus of the paper, but rather are the evidence to stress the need to implement their proposed passive surveillance program in a sustainable manner with the tools that are already available. However, it seems like the authors shift the focus of their paper in presenting their findings. As an example, tables 3, 4 and 5 could be supplemental materials.

R/ We thank reviewer #1 for the constructive and helpful comments. The reviewers' suggestion to standardize a general passive wildlife surveillance scheme for countries with limited resources is an exciting objective. However, we consider that that idea exceeds the scope and intentions of this research. This manuscript aims to communicate our experience of what could be the establishment of a passive epidemiological surveillance system in the wildlife of Costa Rica as a model. This study is a pilot effort with the intention that the scientific community of other countries can visualize what aspects should be considered when aspiring for a surveillance system for wildlife animals. We believe that the current scope of this study can be helpful to countries pursuing this goal.

We also want the manuscript to illustrate aspects such as the type of agents detected and how to approach their diagnosis based on the available diagnostic capacities. This is essential in countries with low income dedicated to epidemiological surveillance of wildlife. In addition, we want to highlight the importance of linkage among human, animal, and wildlife health authorities and higher education institutions under the one health approach. This is based on the urgent need to raise awareness of the benefits of this type of disease monitoring for the countries and its regional and global impact.

Based on this study's limitations, we have recognized the weaknesses in lines: #357-365. However, the proposed program is limited to Costa Rica's resources and infrastructure and does not apply to every country. Therefore, we emphasize that it is important to start evaluating the implementation of these programs in regions where disease surveillance in wildlife is minimal based on experiences like ours. Furthermore, although it is necessary to standardize methods and techniques for monitoring pathogens in wildlife, the development of pilot schemes allows sharing experiences with other programs already installed.

In tune with the European Wildlife Disease Association and OIE, although efforts have been made to expand and unify surveillance programs dedicated to wildlife, significant variations persist. There are still variations in the scope, scale, and capacity for establishing surveillance schemes in many regions. These limitations are mentioned in lines: #372-375.

We are confident that in future studies, we will be able to compare our scheme with other WHMPs in the region to contribute to building these systems in other regions. In addition, other upcoming experiences would help standardize and optimize monitoring systems in the scientific community. Based on the reasons mentioned above and by recognizing the limitations of our study in the manuscript, we believe that the study should maintain the initially conceived structure and leave for future studies the pertinent approach proposed by reviewer 1.

Finally, I found the manuscript difficult to follow. Occasionally, the sentences read awkward (e.g., Lines 50-53), had typos (e.g., Line 55) or wrong terms (e.g., "academy" in Lines 312-

UNA
UNIVERSIDAD NACIONAL
COSTA RICA

314), were grammatically incorrect (e.g., Line 80-82 and Line 95-97), or lacked a clear structure (e.g., Lines 108-112). The authors should do some intense editing to their manuscript, with a more concise language, and, if available, consider asking for English-proof reading services.
R/We apologize for these mistakes. The English language and grammar were revised throughout the text, and the manuscript was improved to make it more concise and fluent.

Reviewer #2:

We would like to thank reviewer #2 for the positive comments

I recommend the use the following categories: metazoan parasites, protozoan parasites.
This was modified as requested

Please, double check the grammar of the text, mainly in the introduction, in general, the English could be a little more fluent. However, I emphasize that this does not affect at all the understanding of the ideas they expose.
The English language was revised throughout the text.

Minor comments:
Line 5: delete of (repeated)
This was corrected

Line 197: change pulmonary parasitic worms to lungworm.
This was corrected

Table 4: change parasitic worms to metazoan parasites.
This was corrected

Sincerely yours,

ALEJANDRO JOSE ALFARO ALARCON (FIRMA)

Firmado digitalmente por ALEJANDRO JOSE ALFARO ALARCON (FIRMA)
Fecha: 2022.08.11 10:14:16 -06'00'

Dr. Alejandro Alfaro-Alarcón PhD.
Department of Anatomic Pathology
National University of Costa Rica
Head of Department
