# Supplementary material for: Passive epidemiological surveillance in wildlife in Costa Rica identifies pathogens of zoonotic and conservation importance
Source: PLoS One. 2022 Sep 26;17(9):e0262063. doi: 10.1371/journal.pone.0262063 (PMC9512195; doi:10.1371/journal.pone.0262063)
Supplement: Attachment — Submitted filename: Response to Reviewers.pdf [file pone.0262063.s005.pdf]

Departamento de Patología
Escuela Medicina Veterinaria
Universidad Nacional
Dr. Alejandro Alfaro, Ph.D.
alejandro.alfaro.alarcon@una.cr

Heredia, September 5, 2021

To

Academic Editor

Reviewers

PLOS ONE

Dear academic editor, dear reviewers,

We would like to thank you all for your observations which will improve our paper. We hope this article would be published soon, the published information could be of great value for wildlife scientists.

Journal Requirements

Please review your reference list to ensure that it is complete and correct. If you have cited papers that have been retracted, please include the rationale for doing so in the manuscript text, or remove these references and replace them with relevant current references. Any changes to the reference list should be mentioned in the rebuttal letter that accompanies your revised manuscript. If you need to cite a retracted article, indicate the article's retracted status in the References list and also include a citation and full reference for the retraction notice.
R/ References were checked:
References #23, #93 and #103 have been replaced with current and relevant references.
References #5, #7, #70 and #73 were removed as they were not relevant.

Reviewer 1

I want to congratulate the authors, not just for the relevance of their paper and the amount of work they did with the implementation of the passive surveillance, but also for the great improvement they made to present their findings, implications and recommendations. I just have a few very minor comments that will be easily corrected.
We would like to thank reviewer #1 for the positive comments.

L34-36: “For instance, 60% (51/85) of the deaths were not associated with an infectious agent. In 67.4% (32/85) of the cases, deaths were not associated with an infectious agent, but an infectious agent was detected.”
These two sentences seem to be repeating the statement, and there seems to be a mistake: 32/85 is 37.6%, not 67.4%. Please check this. Same error in L191.
This was corrected. Line 34-35: “For instance, 60% (51/85) of the deaths were not directly associated with an infectious agent. Though in 37.6% (32/85) of these cases an infectious agent associated or not with disease was detected.”
Line 191: the percentage was corrected.

L37: The acronym “WHMP” is mentioned here, without first stating what does it stand for.
This was corrected.

L84: Consider removing the acronym “LMIC” since it is not used in the rest of the paper, and it is confusing.
This was modified as requested.

Sincerely yours,

ALEJANDRO JOSE ALFARO ALARCON (FIRMA)

Firmado digitalmente por ALEJANDRO JOSE ALFARO ALARCON (FIRMA)
Fecha: 2022.09.05 08:49:43 -06'00'

Dr. Alejandro Alfaro-Alarcón Ph.D.
Departamento de Patología
Escuela de Medicina Veterinaria
Universidad Nacional
alejandro.alfaro.alarcon@una.cr
