# Supplementary material for: Passive epidemiological surveillance in wildlife in Costa Rica identifies pathogens of zoonotic and conservation importance
Source: PLoS One. 2022 Sep 26;17(9):e0262063. doi: 10.1371/journal.pone.0262063 (PMC9512195; doi:10.1371/journal.pone.0262063)
Supplement: Attachment — Submitted filename: PONE-D-21-39637 REV1.docx [file pone.0262063.s002.docx]

PONE-D-21-39637

**General comments**: Initially I would like to congratulate Dr. Alfaro-Alarcon for this manuscript, we know that research efforts to develop studies like the one presented here require a network of motivated institutions and people, this can be evidenced in this manuscript. So please feel proud of this manuscript fruit of his continuous work for almost three years. Congratulations.

This MS is a bit difficult to evaluate, since it could have two different perspectives:

1. Formulation and implementation of the pilot of a Wildlife Health Monitoring Program (WHMP).
2. A study of causes of death and main pathological processes in various species of vertebrates.

My review is in order to analyze this MS with the postulating of WHMP. I strongly believe that this is a manuscript that with some revisions can be used as an example for the installation of a wildlife health surveillance program. Below I respectfully highlight some points that should be considered by you.

I initially consider that some infectious agents should be considered as "primary" for monitoring. I believe that the rabies virus and the flaviviruses would be good pathogens in mammals and influenza and the New Castle virus in birds. These diseases must be carefully selected since they will be the ones that will generate the demand for a national program by the responsible agency. For the other pathogens, I consider appropriate the use of complementary techniques based on histopathological analysis (CDV, bacterial).

In order to replicate this program, I felt a huge lack of a flowchart in the methodology describing each of the institutions with their respective activities. I understand that all the necropsies were carried out by the veterinary pathologists of the university involved in this study, but in order to improve the efficiency and effectiveness of this program, they do not believe that it would be better if the necropsies and sample collection were carried out directly in the institution (in this way the freezing of corpses would be avoided, which improves the histopathological evaluation).

The carcasses that were found in the "forest" can really provide insight into the pathogens that are circulating in the ecosystem. However, animals that have entered a rescue center may be exposed to pathogens that are stabilized on the installations. Considering this, was there any inclusion criteria for animals from rescue centers, for example, animals that arrived dead or that lasted a maximum of 24 hours in the institution?

I think that seeking to create a diagnostic algorithm within the wildlife health program that you propose, perhaps a classification of causes of death like this could be better:

- Death associated with an infectious agent.
- Death not associated with an infectious agent, with a pre-existing infectious disease.
- Death not associated with an infectious agent, with a molecular diagnosis of an infectious pathogen.
- Death not associated with an infectious agent.
- Undetermined death.

With these categories you could see which species may be sensitive (which would be ideal for passive surveillance by acting as sentinels) and which are resistant (which would be ideal for serological inquiries) to the selected pathogens.

I believe that in order to establish a wildlife health surveillance program, particularly in countries with limited resources, it is important to prioritize efforts, to those pathogens of greatest importance to human health (based on epidemiological data from the Health Agency), as well as for biodiversity conservation (based on wildlife mortality data).

I confess that when presenting absolute and proportional results I find it interesting to use the following form: X% (n/N). It is not something that they should replicate but if you find it interesting it would be great.

**Introduction:** Despite being a somewhat extensive introduction (for me), I consider that addressing the points that will be dealt with in the manuscript, I make some specific comments in the minor revision section.

**MM**: If you accept my comments please describe the WHMP better, I have no objection to the proposed diagnostic methodologies. I am sure that you are quite judicious about it.

**Results and discussion**: As things change in the methodology you will see that the results can be written in another way, I will make two suggestions for tables, and please when you are writing the results do not forget that the focus of the manuscript is not pathology, it is the implementation of the WHMP. Please, be a little more summarized in the pathological aspects (S2 table is enough) and increase the information about the program. I make this same comment for discussion. Remember that discussing whether or not the non-infectious cause of death is frequent, for the purpose of this manuscript, may be secondary. But discussing the limitations, benefits, diagnostic techniques, costs, can be much more interesting.

**Tables**:

If the authors consider my suggestions, Table 1 should include the absolute and relative values of the causes of death for each of the taxonomic groups, that is:

| **Cause of Death / Taxon** | **DAIA** | **DNAIA-PD** | **DNAIA-ADT** | **DNAIA** | **UD** |
| --- | --- | --- | --- | --- | --- |
| **Mammals** | % (n/N) | % (n/N) | % (n/N) | % (n/N) | % (n/N) |
| **Carnivores** | % (n/N) | % (n/N) | % (n/N) | % (n/N) | % (n/N) |
| **Birds** | % (n/N) | % (n/N) | % (n/N) | % (n/N) | % (n/N) |
| **Pelecaniformes** | % (n/N) | % (n/N) | % (n/N) | % (n/N) | % (n/N) |

DAIA= Death associated with an infectious agent; DNAIA-PD= Death not associated with an infectious agent, with a pre-existing infectious disease; DNAIA-ADT= Death not associated with an infectious agent, with an additional diagnostic tool; DNAIA= Death not associated with an infectious agent; UD= Undetermined death.

Table 2 would include the pathogens diagnosed with the respective techniques.

S1. Table: please add the scientific name of all specimens.

**Figures**: please homogenize the formatting of the figures, if you are going to place a scale bar, place them in all of them, otherwise, remove them in all of them. In the case of metazoan parasites, describe if they were identified morphologically and/or molecularly, otherwise if they were by tissue characteristics, mention which guide was used (eg, Gardiner or Chitwood), and describe the order and/or the superfamily, and later if they want they can mention the potential genus (explaining that this genus is postulated by previous accounts in the species or close taxonomic group). I confess that I feel a lack of macroscopic photos. but they are not necessary

Figure 1.

A. To exemplify the cases of CDV in none of the animals were inclusion corpuscles observed? If these were observed, my suggestion is to place a photo of one of them. The caption provided describes the image well.

B. The inflammatory component is not fully observed in the microphotograph, I confess that it was a bit difficult for me to show the granulomatous component (If it is possible to take another microphotograph where it is more evident, it is highly recommended), otherwise, I recommend modifying the legend of this Photo.

C. In this microphotography it is not possible to show the Trypanozoma amastigotes, the myocardial inflammatory process is well described in the legend, my suggestion is to place a microphotograph at a higher magnification (eg 400x or 1000x) only of the amastigote, if you have a field where are inside the myocardial fiber would be great.

D. I strongly recommend taking a photomicrograph that expresses the necrotizing component of hepatitis, otherwise you should change the caption on this photo.

E. In my opinion if you don't have a potential cause for this injury, I recommend removing it.

F. I recommend removing this photo, since initially this is not a finding associated with an infectious component.

Figure 2

A. Surely you analyzed it, but to exemplify toxoplasmosis I think a microphotograph of a liver with tachyzoites and the lobular inflammatory and necrotizing process would be much more illustrative. If this was not possible, a pity, the photograph and the legend are fine, just a change, according to Dubey in the latest updates on toxoplasmosis, the term that should be used is no longer pseudocyst, now tissue cyst should be used.

B. They should be proud of their histotechnician, those histological cuts are excellent!!!! I only recommend not mentioning the type of inflammatory cell since at this magnification it is not possible to identify them (even zooming in on the image).

C. Excellent microphotography, the marvelous lesion!!! (poor primate), just a recommendation, we know that by culture you are sure of the bacteria, but in the histopathological slide my suggestion is to report with intralesional bacteria (*Klebsiella pneumoniae* by culture), if you want to place an insert I would suggest placing a photo of it histological gram field. otherwise I would remove the inset.

D. These lungworms were identified morphologically and/or by molecular techniques, or solely by the characteristics of the worm in the tissue. if it was solely for tissue characteristics please don't use gender, as we know that morphological keys are only suitable up to order or superfamily. It would be interesting to mention that in this cut they have both males and females (many more cuts of the female), since this helps to understand the cycle of this beautiful nematode. As in photo B, due to the magnification, it is not possible to identify the inflammatory cells.

E. Same previous comment on classification, additionally in that photo the nematodes are not illustrated (in the same way as in the other microphotographs).

F. This microphotograph is not very illustrative, the different layers of the tissue are not observed, in my opinion this photo could be removed.

G. Same previous comment on classification, additionally I want to tell you that this microphotograph is very pretty. I suggest that in the legend they mention the fibroplasia component (which is evident) and that it helps the morphological diagnosis of this Nodular and sclerosing gastritis condition.

H. The photo is very good, but it is nothing more than a photo of a sarcocystis, if it were associated with an inflammatory process, I would recommend including it in the manuscript, otherwise I recommend removing it.

I. Excellent microphotography, I don't know if this condition is reported in that particular species, congratulations.

Minor comments

Line 28: please add space in “the detection”.

Line 30: please remove a space (there are double spaced) between words “a solid”

Line 47-53: Dear author, I would like to better understand your statement, it represents a burden for society in what sense (economic due to the overload of health systems, due to treatment costs, etc.). In the manuscripts that you are citing there is interesting material for you to formulate a sentence with greater impact. I fully understand that the pandemic caused by SARS-CoV 2 can seduce us to cite it as an example; however there are other diseases that can serve as an example and can generate impacts for both humans and wildlife (e.g., yellow fever, tuberculosis, rabies, influenza, West Nile Virus (interesting example in your specific case :D) etc.). Respectfully, I encouraged you to write a new sentence.

Line 83-87: Dear authors, I considered that it would be interesting to add some additional information on the programs currently installed in some Latin American countries where wildlife is considered within the national surveillance programs (eg, non-human primates in yellow fever in Brazil [https://bvsms.saude.gov.br/bvs/publicacoes/guia_vigilancia_epizootias_primatas_entomologia.pdf]; in Argentina, game species are used to monitor some diseases such as trichinellosis [https://www.argentina.gob.ar/sites/default/files/informe_trichinellosis_2010_2019.pdf]; unfortunately those are the only two programs that I know of but with this information you could mention that there are some national programs installed and working perfectly where wild animals are used as sentinels for surveillance of some diseases).

Line 139: change histological by histopathological.

Line 165: typing error “sender”

Line 166-167: I believe that in order to successfully replicate this study it would be interesting if the authors describe the criteria used to classify the age-range of the specimens.

Line 184-193: Does the number of 199 include all the organs evaluated in the pathology?

**Final comments**: Once again, I consider that this manuscript should be published since it brings a potential benefit to the scientific community that the readers of this prestigious journal will surely take advantage of. Obviously, the manuscript as it is at this moment needs some changes that I have no doubt that Dr. Alfaro-Alarcon and his team can make without any difficulty. I thank you for the opportunity to evaluate this important manuscript and I send you my best energies so that you can send it back with the suggested comments. Again, I remind you that I made this evaluation with great respect and my greatest critical sense so that you have a highly competitive manuscript.
